# Home blood pressure monitoring: methodology, clinical relevance and practical application: a 2021 position paper by the Working Group on Blood Pressure Monitoring and Cardiovascular Variability of the European Society of Hypertension

**DOI:** 10.1097/HJH.0000000000002922

**Published:** 2021-07-26

**Authors:** Gianfranco Parati, George S. Stergiou, Grzegorz Bilo, Anastasios Kollias, Martino Pengo, Juan Eugenio Ochoa, Rajiv Agarwal, Kei Asayama, Roland Asmar, Michel Burnier, Alejandro De La Sierra, Cristina Giannattasio, Philippe Gosse, Geoffrey Head, Satoshi Hoshide, Yutaka Imai, Kazuomi Kario, Yan Li, Efstathios Manios, Jonathan Mant, Richard J. McManus, Thomas Mengden, Anastasia S. Mihailidou, Paul Muntner, Martin Myers, Teemu Niiranen, Angeliki Ntineri, Eoin O’Brien, José Andres Octavio, Takayoshi Ohkubo, Stefano Omboni, Paul Padfield, Paolo Palatini, Dario Pellegrini, Nicolas Postel-Vinay, Agustin J. Ramirez, James E. Sharman, Andrew Shennan, Egle Silva, Jirar Topouchian, Camilla Torlasco, Ji Guang Wang, Michael A. Weber, Paul K. Whelton, William B. White, Giuseppe Mancia

**Affiliations:** a Istituto Auxologico Italiano, IRCCS, San Luca Hospital, Department of Cardiovascular Neural and Metabolic Sciences; b Department of Medicine and Surgery, University of Milano-Bicocca, Milan, Italy; c Hypertension Center STRIDE-7, National and Kapodistrian University of Athens, School of Medicine, Third Department of Medicine, Sotiria Hospital, Athens, Greece; d Division of Nephrology, Department of Medicine, Indiana University School of Medicine and Richard L. Roudebush Veterans Administration Medical Center, Indianapolis, Indiana, USA; e Department of Hygiene and Public Health, Teikyo University School of Medicine, Tokyo, Japan; f Research Unit Hypertension and Cardiovascular Epidemiology, KU Leuven Department of Cardiovascular Sciences, University of Leuven, Leuven, Belgium; g Tohoku Institute for the Management of Blood Pressure, Sendai, Japan; h Foundation-Medical Research Institutes, Geneva; i Service of Nephrology and Hypertension, University Hospital, Lausanne, Switzerland; j Hypertension Unit, Department of Internal Medicine, Hospital Mútua Terrassa, University of Barcelona, Barcelona, Spain; k Cardiology IV, ‘A. De Gasperis” Department, ASTT GOM Niguarda Ca’ Granda; l Cardiology/Hypertension Unit Saint André Hospital. University Hospital of Borfeaux, France; m Baker Heart and Diabetes Institute, Melbourne, Victoria, Australia; n Division of Cardiovascular Medicine, Department of Medicine, Jichi Medical University School of Medicine, Tochigi, Japan; o Shanghai Institute of Hypertension, Ruijin Hospital, Shanghai Jiaotong University School of Medicine, Shanghai, China; p Department of Clinical Therapeutics, National and Kapodistrian University of Athens, School of Medicine, Alexandra Hospital, Athens, Greece; q Primary Care Unit, Department of Public Health & Primary Care, University of Cambridge, Cambridge, UK; r Nuffield Department of Primary Care Health Sciences, University of Oxford, Oxford, UK; s Kerckhoff Clinic, Rehabilitation, ESH Excellence Centre, Bad Nauheim, Germany; t Department of Cardiology and Kolling Institute, Royal North Shore Hospital, St Leonards Faculty of Medicine and Health Sciences, Macquarie University, Sydney, Australia; u Hypertension Research Center, Department of Epidemiology, School of Public Health, University of Alabama at Birmingham, Birmingham, Alabama, USA; v Schulich Heart Program, Sunnybrook Health Sciences Centre and Department of Medicine, University of Toronto, Toronto, Canada; w Department of Medicine, Turku University Hospital and University of Turku; x Finnish Institute for Health and Welfare, Helsinki, Finland; y The Conway Institute, University College Dublin, Dublin, Ireland; z Experimental Cardiology, Department of Tropical Medicine Institute, Universidad Central de Venezuela, Venezuela; aa Clinical Research Unit, Italian Institute of Telemedicine, Varese, Italy; ab Department of Cardiology, Sechenov First Moscow State Medical University, Moscow, Russian Federation; ac Department of Medical Sciences, University of Edinburgh, Edinburgh, UK; ad Studium Patavinum, Department of Medicine. University of Padova, Padua; ae Cardiovascular Department, ASST Papa Giovanni XXIII, Bergamo, Italy; af Hypertension Unit, European Georges Pompidou Hospital, Paris, France; ag Arterial Hypertension and Metabolic Unit, University Hospital, Fundacion Favaloro, Argentina; ah Menzies Institute for Medical Research, College of Health and Medicine, University of Tasmania, Hobart, Australia; ai Department of Women and Children's Health, School of Life Course Sciences, FoLSM, Kings College London, UK; aj Research Institute of Cardiovascular Diseases of the University of Zulia, Venezuelan Foundation of Arterial Hypertension. Maracaibo, Venezuela; ak Diagnosis and Therapeutic Center, Paris-Descartes University, AP-HP, Hotel Dieu, Paris, France; al Division of Cardiovascular Medicine, Downstate College of Medicine, State University of New York, Brooklyn, New York, USA; am Department of Epidemiology, Tulane University, School of Public Health and Tropical Medicine, New Orleans, Lousiana; an Cardiology Center, University of Connecticut School of Medicine, Farmington, Connecticut, USA; ao University Milano-Bicocca, Milan, Italy

**Keywords:** blood pressure measurement, cardiovascular disease, cardiovascular risk, home blood pressure monitoring, hypertension, prevention and control

## Abstract

The present paper provides an update of previous recommendations on Home Blood Pressure Monitoring from the European Society of Hypertension (ESH) Working Group on Blood Pressure Monitoring and Cardiovascular Variability sequentially published in years 2000, 2008 and 2010. This update has taken into account new evidence in this field, including a recent statement by the American Heart association, as well as technological developments, which have occurred over the past 20 years. The present document has been developed by the same ESH Working Group with inputs from an international team of experts, and has been endorsed by the ESH.

## 1. INTRODUCTION

Arterial hypertension is one of the principal cardiovascular risk factors, and still represents a largely unmet public health challenge given its close association with mortality and morbidity globally because of cardiovascular, cerebrovascular and kidney disease-related complications [1,2]. The current suboptimal management of hypertension might be in part related to the limitations of using only office blood pressure (BP), which has led to increasing use of out-of-office BP [1,2]. Adoption of home BP monitoring (HBPM), in particular, has had an exponential growth, favoured by technological progress, which has led to the availability of small, accurate, user-friendly and relatively inexpensive BP monitoring devices. The present paper provides an update of previous recommendations from the ESH Working Group on BP Monitoring and Cardiovascular Variability (BPMCVV) sequentially published in years 2000 [3], 2008 [4] and 2010 [5] and from AHA [6].

## 2. PURPOSE AND SCOPE

Hypertension guidelines have to deal with a large number of complex issues, which limits the space for a detailed discussion on practical aspects of BP measurement. This has been the case for HBPM as well, which is recommended in recent hypertension guidelines but without detailed instructions on its practical application. These instructions are provided in the present manuscript, together with an update on the emerging technologies in this field in order to provide healthcare professionals with guidance that details the appropriate use of contemporary HBPM in clinical practice and research [1,2,6–12].

## 3. WHAT IS NEW IN THE 2021 POSITION PAPER

Compared with the 2008/2010 documents, the evidence accumulated over the past 12 years has allowed for modification of a number of previous statements or recommendations. This is summarized in Table 1.

**TABLE 1:** Main changes between 2008/2010 and 2021 HBPM position papers

Topic	2008/2010	2021
Cuff size	Different cuff sizes are recommended for patients with different arm circumferences	Cuff choice should consider patient's arm size but should also be based on the instructions by the manufacturer, based on evidence from validation studies. The use of wide-range cuffs with automated devices may be particularly useful
Clinical validation protocols	Several validation protocols are recommended (BHS, AAMI, ESH International Protocol)	The 2018 Universal Standard AAMI/ESH/ISO is recommended for all new validation studies
Cuffless devices	(not mentioned)	Now available, they should undergo thorough clinical validation (appropriate validation protocol by AAMI/ESH/ISO under preparation) before being recommended for performing HBPM
Information on validated devices	DABL	STRIDE BP, www.stridebp.org
	BIHS	BIHS, www.bihsoc.org/bp-monitors
	VDL	US BP VDL, www.validatebp.org
	Hypertension Canada	Hypertension Canada, www.hypertension.ca/bpdevices
	Deutsche Hochdruckliga	Deutsche Hochdruckliga, www.hochdruckliga.de/betroffene/blutdruckmessgeraete-mit-pruefsiegel
	JSH	JSH, www.jpnsh.jp/com_ac_wg1.html
	MEDAVAL	
Preferred devices	(not mentioned)	Preferred HBPM devices (www.stridebp.org) now specified as:: upper arm cuff devices with at least one STRIDE BP approved validation study published in the last 10 years and using a recent protocol (AAMI/ESH/ISO 2018; ANSI/AAMI/ISO 2013 or 2009; ESH-IP 2010); being in use for less than 4 years [13]; and equipped with storage/connectivity facilities for objective reporting of readings
Monitoring schedule and interpretation	3--7 days monitoring schedule, with BP values measured on the first monitoring day to be discarded	3--7 days monitoring schedule, with 2 measures taken in the morning and evening. Discarding the first day may have an effect on a 3-day schedule, but appears to have minimal impact with more monitoring days
Diagnostic thresholds	Threshold for hypertension at least 135/85 mmHg for SBP/DBP less than 130/80 mmHg normal HBP	The threshold for ESC/ESH hypertension diagnosis is 135/85 mmHg (corresponding to 140/90 mmHg of clinic BP in the ESC--ESH hypertension guidelines). HBP of 130/80 mmHg may correspond to 130/80 mmHg clinic BP threshold for grade I hypertension used in ACC/AHA guidelines
Therapeutic targets	No recommendations	Systolic HBP between 125 and 135 mmHg for most people. Diastolic HBP between 70 and 80 mmHg as a reasonable goal. In the frail very elderly, slightly higher systolic HBP might be the preferred target (suggested in the 140–150 mmHg range but more evidence is needed), while avoiding excessive reductions of diastolic HBP.
Children	Few suggestions regarding when and how frequently HBPM should be measured in children	Preliminary evidence supports use of a HBPM schedule similar to that recommended for adults
Pregnancy	HBPM should be performed with the woman seated or lying on her side at a 45° angle	The sitting position appears to be appropriate for HBPM during pregnancy. The same 3--7 days monitoring schedule recommended. Concerning BP threshsolds: in unselected women HBP = clinic BP [14]
Chronic kidney disease on dialysis (ESKD)	No recommended schedule	HBP should be measured twice daily, at bedtime and on waking up, after the midweek dialysis for 4 days
Arrhythmias	In patients with frequent or persistent arrhythmias, HBPM should not be used as the sole diagnostic tool	With HBPM, automated devices should be preferred to auscultatory devices and used even in the presence of atrial fibrillation (AF) (with controlled ventricular rate) triplicate measurements could be useful because of increased beat-to-beat variability. In the case of uncontrolled tachyarrhythmias automated devices may provide inaccurate readings (a debated issue) [15] AF detecting algorithm during automated HBPM might be useful for early detection of asymptomatic AF in elderly individuals with hypertension
Nocturnal HBPM	Lack of night recordings as a limitation of HBPM	With technological development of devices, nocturnal HBPM is feasible and appears to be a promising alternative to ABPM for the evaluation of sleep BP
Home BP variability	Not mentioned	Home BP variability is an independent outcome predictor but the current evidence is insufficient to support its application in clinical practice

AAMI, Association for the Advancement of Medical Instrumentation; ACC, American College of Cardiology; AHA, American Heart Association; BHS, British Hypertension Society; ESC, European Society of Cardiology; ESH, European Society of Hypertension; HBPM, home blood pressure monitoring.

## 4. METHODOLOGY OF THE POSITION PAPER

The present recommendations are based on evidence provided by papers published until January 2021, from which a draft prepared by a writing committee (G.P., G.S.S., A.K., E.O.M., G.B. and M.P.) was circulated among all the authors of this document who reviewed and approved its final version.

## 5. DEFINITIONS AND TERMINOLOGY: WHAT IS HBPM?

HBPM has become the universally used term to define a procedure by which an individual self-measures BP noninvasively in his/her home [16]. To ensure standardization of HBPM, the conditions summarized in Box 1 should be fulfilled.

Box 1 **HBPM characteristic features**

- Measurements should take place in individuals’ home (pharmacy or workplace BP measurements are not home measurements).
- Measurements should be self-performed, with assistance by family member or others in case of physical or cognitive limitations or in children. A measurement taken by healthcare personnel visiting patient's home is not HBPM.
- Individuals should be instructed on the appropriate methodology, as well as on the measurement schedule, best if during a structured hypertension teaching program.
- BP values should be reported as a downloadable electronic log maintained in the monitor memory or in directly connected mobile phone, or tele-transmitted for the physician's review. If this is not possible, then a paper form should be provided for patients to report their readings.
- The next recommended step is discussion between the individual and the healthcare provider to determine an appropriate management plan.

## 6. HBPM: ADVANTAGES AND LIMITATIONS

### 6.1 Reproducibility (Box 2) (more details in the online supplemental file S6.1)

The reproducibility of any BP measurement method improves by increasing the number of BP readings, and thus one of the advantages of HBPM is the larger number of readings that can be obtained compared with office BP (OBP) measurement [17,18]. Although head-to-head comparison studies are scarce, a review of the studies on test--retest correlation coefficients and standard deviation of differences between repeated home and ambulatory BP measurements suggests a similar reproducibility of the two methods, and a better reproducibility of both as compared with OBP [18,19], with some studies suggesting a better reproducibility of HBPM than ambulatory BP monitoring (ABPM) [20,21].

Box 2

- HBPM has better reproducibility than OBP.
- HBPM has reproducibility that is comparable to ABPM.
- The better reproducibility of HBPM vs. OBP, both in short-term and long-term, is at least in part related to the inclusion of a higher number of readings in its assessment and to a better standardized measurement condition.

### 6.2 Diagnostic ability (Box 3)

#### 6.2.1 Diagnostic accuracy and identification of masked and white-coat hypertension (more details in the online supplemental file S6.2.1)

Studies [1,2,7–9] comparing HBPM with ABPM in detecting masked hypertension report that in about half of the patients diagnosed with masked hypertension, this phenomenon is present with both ABPM and HBPM (dual masked hypertension), whereas in the remainder, the diagnosis is only documented with one of the two BP measurement methods [22–25]. In outcome studies, individuals with masked hypertension identified by only HBPM or ABPM have an intermediate level of cardiovascular disease (CVD) risk between those identified by both methods as normotensives and those as sustained hypertensive patients. Recently, it has been shown that masked hypertension diagnosed by ABPM and not by HBPM, or the reverse, is not uncommon, with age being the most important determinant of isolated ambulatory or home masked hypertension, with the former being more common in younger participants and the latter in older ones [23].

Box 3 **Diagnostic capability of HBPM**

- Both HBPM and ABPM have been recommended for wide use by recent United States and European Hypertension guidelines.
- HBPM and ABPM provide similar, though not identical information on BP and appear to be complementary diagnostic methods.
- In hypertension diagnosis, HBPM has higher specificity and negative-predictive value, and lower sensitivity and positive-predictive value than ABPM, in particular for the diagnosis of white-coat and masked hypertension.
- In a high proportion of individuals, masked or white-coat hypertension are identified only by HBPM or only by ABPM. In outcome studies, these individuals have intermediate CVD risk between those identified by both methods as normotensives and those as sustained hypertensive patients.
- Studies assessing morning BP showed similar results with HBPM or ABPM (see online supplement S.2.1).

### 6.3 Prognostic value of HBPM

#### 6.3.1 Hypertension-mediated organ damage (Box 4) (more details in the online supplemental file S6.3.1)

##### Cardiac damage

Left ventricular hypertrophy (LVH) is the most widely used marker of cardiac damage in hypertension. In a meta-analysis of 14 studies, echocardiographically measured left ventricular mass index (LVMI) correlated with home SBP/DBP with pooled correlation coefficients of 0.46/0.28, respectively. These correlations were similar to that with ABP (0.37/0.26, 9 studies) and superior to that with OBP (0.23/0.19, 10 studies) [26]. Statistically significant correlations of HBP with LVMI have also been reported in other studies, not included in this meta-analysis [27–31].

Box 4 **HBPM and target organ damage**

- LVMI is more closely associated with HBP than with OBP, and this association is as strong as that observed with ABP.
- Some studies suggest that HBP is more closely associated with carotid atherosclerosis than OBP and ABP, whereas no difference between methods was found for PWV.
- Although HBP generally correlates with arterial and kidney hypertension-mediated organ damage (HMOD), its superiority over OBP in this regard is not unequivocally supported by available evidence.
- Masked hypertension detected by HBP was characterized by more pronounced organ damage than normotension, similar to findings in sustained hypertension.

##### Vascular damage

A meta-analysis of four studies found only weak correlations of home and office SBP/DBP with carotid intima--media thickness (cIMT) with no evident differences between the two BP-measuring methods [26]. In contrast, the Ohasama study reported that HBP is more closely associated with carotid atherosclerosis (increased cIMT or presence of plaques) than OBP and ABP. In this study, HBP was also a better predictor of silent cerebrovascular disease than OBP [32].

##### Kidney damage

A systematic review reported similar correlations between urinary protein excretion and HBP and OBP values based on evidence from two studies [26]. There was a similar finding in a study by Matsui *et al.*[28]. On the other hand, the ADVANCED-J study in diabetic patients and the J-HOP study showed that HBP was more closely associated with urinary albumin--creatinine excretion ratio (UACR) than OBP [27,33]. In the latter study, HBP appeared to be superior to ABPM in this regard [27]. In another study from Indianapolis, urine protein excretion was related to office, home and ABP; OBP was weakly related to urine protein excretion, HBP more strongly, and ABP showed the strongest association [34].

#### 6.3.2 Adverse health outcomes (Box 5) (more details in the online supplemental file S6.3.2)

Evidence regarding the relationship between HBP and clinical outcome has been summarized in several papers including the previous ESH HBPM guideline [5], two systematic reviews [35,36] and, in a recent narrative review [37]. Overall, solid evidence demonstrates that HBP is associated with CVD risk in community-based cohorts (Ohasama study, Kahoku study, PAMELA study and Didima Study), as well as in clinic-based cohorts (Table S1). Moreover, HBP appears to be superior to OBP in predicting outcomes: in the meta-analysis by Ward *et al.* HBP was associated with higher odds ratios (OR) than OBP for total mortality (ORs per 10 mmHg SBP/DBP increase: 1.14/1.10 vs. 1.07/1.02 for HBP and OBP, respectively), cardiovascular mortality (1.29/1.17 vs. 1.15/1.07) and cardiovascular events (1.14/1.13 vs. 1.10/1.07), with similar findings reported in a second meta-analysis [36]. Also when HBP and OBP were mutually adjusted, only the former remained a significant predictor of outcomes [35]. Similar data have been reported for major cardiovascular events in the IDHOCO database and in the HONEST Study [38,39]. In the former study, HBP was a stronger predictor of outcome than OBP in treated individuals while there were no clear differences between the two methods in untreated ones [38]. Current evidence does not clearly support either HBP or ABP as the superior method for predicting outcome [40]. In the PAMELA study, ABP did not add prognostic information when the data from OBP and HBP were combined; however, only two HBP readings on a single day were obtained in this study [41]. A systematic review summarized studies that assessed both HBP and ABP in terms of outcome prediction. Direct comparisons of hazard ratios did not reveal consistent differences between the two methods, and in the few studies where mutual adjustment was performed, only one indicated clear superiority of ABP in this regard [42]. In a recent paper using data from the PAMELA population, addition of out-of-office SBP or DBP to OBP improved cardiovascular and all-cause mortality prediction. The improvement was more consistent when HBP rather than ABP was added to OBP and, compared with HBP with OBP, no better prediction was found when addition was extended to 24-h ABP. With all additions, however, the improvement was quantitatively modest [43]. A summary of prospective studies linking HBPM to outcome is provided in Table S1, in the online supplement.

Box 5 **HBPM and OUTCOME**

- HBP is associated with cardiovascular risk in community-based as well as in clinic-based cohorts.
- HBP appears superior to OBP in predicting outcomes.
- HBP is more closely associated with both stroke and end-stage kidney disease than OBP. In case of coronary events, this stronger association may be restricted to untreated individuals.
- At present, there is no convincing evidence suggesting superiority of either HBP or ABP in predicting outcome.

### 6.4 Improving adherence to treatment and BP control (Box 6)

#### 6.4.1 Improving adherence to treatment (more details in the online supplemental file S6.4.1)

Low adherence to antihypertensive medications is the most common cause of treatment resistance [44] and it is associated with an increased risk of cardiovascular events [45]. Strategies focused on improving adherence are complex and related to patient's behaviour and education, physician attitude, complexity of drug regimen and other healthcare supportive measures [46]. As HBPM requires active cooperation by the patient, it may be particularly effective in favourably affecting patients’ perceptions of their hypertension, thereby encouraging them to be compliant with lifestyle modifications and antihypertensive therapy. Although HBPM, as an isolated intervention, has been associated with a significant increase in the number of pills taken per week [47,48], establishing the specific contribution of HBPM to treatment adherence has not been easy as in most studies, HBPM has been used in combination with other interventions, such as telemonitoring, patient counselling, additional education or medication reminders [49]. A meta-analysis of 28 trials with more than 7000 participants revealed a significant modest positive effect of HBPM (isolated or associated to other co-interventions) on medication adherence when measured objectively by pill count or electronic monitoring [49,50] The question on whether improving treatment adherence is more effective when based on HBPM alone, on HBPM combined with telemonitoring and with feedback to patients by their doctor between visits, or when based exclusively on BP determination during office visits is currently being addressed [51–53].

Box 6 **Improving adherence to treatment and BP control with HBPM**

- HBPM is recommended as a means to increase long-term treatment adherence and persistence and to stimulate other lifestyle changes.
- More research is needed to establish whether improving treatment adherence is more effective when based on HBPM alone, on HBPM combined with telemonitoring and with feedback to patients by their doctor between visits, or when based exclusively on BP determination during office visits.
- A number of studies have shown that treatment titration based on HBPM is associated with better BP control rates than when based on OBP.
- HBPM is more effective in improving BP control when combined with education and counselling.

#### 6.4.2 Improving BP control during the long-term follow-up

Some studies (i.e. TASMINH2 and TASMIN-SR studies) have shown that patients can also use HBP to titrate their own antihypertensive medication successfully, which translates into improved BP control rates [54,55]. In a meta-analysis, HBPM was associated with less therapeutic inertia; physicians were more likely to change BP medications whenever HBP was found to be elevated [56]. Recent meta-analyses have shown that self-monitoring alone is not associated with lower BP values or with better BP control rates, but in conjunction with co-interventions (including systematic medication titration by doctors, pharmacists, or patients; education; or lifestyle counselling), it leads to clinically significant BP reduction, which persists for at least 12 months [57]. In consideration of this evidence, it is recommended that implementation of self-monitoring in hypertension should be accompanied by such co-interventions in all treated hypertensive patients [58,59]. HBPM can also contribute to maintain hypertension control at the time of seasonal BP changes. Although ABPM might be regarded as the most suitable method for the identification of seasonal BP changes (reflecting the effect of indoor and outdoor conditions), a recent meta-analysis showed that HBPM may also identify these changes [60]. Thus, HBPM can also be used to identify hypertensive individuals with excessive seasonal BP changes, and effectively titrate antihypertensive treatment [61]. Evidence from studies implementing HBPM has also indicated that the prevalence of masked hypertension is higher in seasons other than summer also showing a significant association between morning home DBP and HMOD in winter [62].

### 6.5 Possible difficulties with HBPM use in clinical practice (Box 7)

Despite its advantages, the clinical application and the accuracy of HBPM may be limited by certain conditions related either to the individual [63–66], to the procedure itself [4,67], to the oscillometric technique for BP measurement or to cuff-related issues [4,65,68–74].

Box 7 **Difficulties with HBPM use**

**Difficulties related to HBPM procedure**

- Need of patient training (short-lasting for automated devices)
- Possible use of inaccurate devices
- Limited reliability of BP values reported by patients
- Induction of anxiety, resulting in higher BP levels and excessive number of measurements
- Inappropriate treatment changes made by patients based on of casual home measurements without doctor's guidance.
- Normality thresholds and therapeutic targets still to be defined
- Lack of night-time readings with most available devices

**Difficulties related to the oscillometric BP measurement technique**

- A number of devices still inaccurate, although the situation is improving
- Oscillometric technique fails in some individuals and such patients should be identified
- New wearable oscillometric devices still need extensive validation

**Difficulties related to individuals**

- Children
  ∘ Limited research in clinical application
  ∘ Uncertain reference values
  ∘ Arterial compliance and cuff size-related issues
  ∘ Need for specific validation of oscillometric devices
  ∘ Few devices validated (see www.stridebp.org/bp-monitors)
  ∘ HBPM schedule not easily followed
  ∘ Uncertain diagnostic role
- Elderly
  ∘ Increased BP variability
  ∘ Limited patient's performance/compliance
- Obese people
  ∘ Need of cuffs with adequate size and shape
- Arrhythmias
  ∘ Issues with BP measurement accuracy
  ∘ Possible inaccuracy of built-in software for arrhythmia detection
  ∘ Need of repeated measurements
- Pregnancy
  ∘ Need for specific validation of oscillometric devices
  ∘ Few devices validated (see www.stridebp.org/bp-monitors)
  ∘ BP underestimation in preeclampsia
  ∘ Uncertainty of BP thresholds and treatment targets
  ∘ Uncertainty of efficacy and place in care pathways
- End-stage kidney disease and diabetes
  ∘ Reduced accuracy of the oscillometric devices because of arterial stiffness typical of these conditions
  ∘ Presence of arterio-venous fistula may affect measurement accuracy

## 7. TECHNOLOGY OF HBP MONITORS

### 7.1 Cuff-based devices

#### 7.1.1 Types of cuff-based devices for HBPM (more details in the online supplemental file S7.1.1)

Several techniques for measuring BP are used by HBPM devices. The most widely used techniques (auscultatory and oscillometric) are described below, with mention of new perspectives for wearable devices offered by progress in technology.

##### Auscultatory method

The manual auscultatory method involves the detection of the Korotkoff sounds and is based on the use of aneroid, mercury (wherever available) or hybrid devices. This approach requires skills, good hearing, substantial patient training and regular calibration in case of aneroid devices [1,2,12,75]. Very few devices incorporate microphones or specific sensors to perform automatic auscultatory (microphonic) measurement of BP with less user interference. Overall, the auscultatory method is not currently recommended for HBPM, due both to its difficult implementation and poor patients’ performance.

##### Oscillometric method

Most automated or semi-automated electronic devices for BP measurement use the oscillometric method [76]. Each device has its own proprietary algorithm to calculate BP from the collected oscillometric signal. Most of these devices acquire data for measurements during cuff deflation whereas some do this during cuff inflation. As each device has its own specific proprietary algorithm and technical characteristics, the measurement accuracy of one device cannot be extrapolated to another, even if produced by the same manufacturer. Moreover, as the cuff with the oscillometric method is used not only to obtain arterial occlusion but also as a sensor to collect the oscillometric signal, experts agree that each oscillometric device must be used only with its own specific cuff(s) as provided by the manufacturer. Therefore, HBPM devices must be considered as the combination of a device and its accompanying cuff(s), whereas the cuff size and type rules, which apply for the auscultatory method may not be applicable to the oscillometric approach. Electronic oscillometric devices require less training and are user-friendly, relatively inexpensive and generally not affected by observer bias if used correctly. These devices, must meet the requirements of national and international regulatory bodies for safety of medical devices, such as the Food and Drug Administration (FDA) in the United States, and the CE (Conformité Européenne) labeling in Europe but it is recommended to use only devices that have also undergone independent validation for accuracy and passed the criteria of established validation protocols (see paragraph on Clinical Validation). A list of validated HBPM devices can be found, among others, on the British and Irish Hypertension Society and STRIDE BP websites (www.stridebp.org).

###### Measurements at different sites

Automatic oscillometric devices have been designed to measure BP at different arterial sites. The most commonly used (and recommended) ones are those measuring BP at the upper arm (brachial artery) level and to a lesser extent those measuring BP at the wrist (radial artery) level. Devices that measure BP at the finger level are not recommended [77].

**Wrist cuff-based devices** are popular among patients as measurement is readily obtained without the need to remove clothing, and can be useful in extreme obesity when even extra large cuff is too smal [78]. These devices are individual to limitations, such as distal measurement site and limb position. Even though several automated wrist devices have successfully passed international validation protocols in a laboratory setting, they are considered more prone to errors than the upper arm devices in real-life conditions [79]. Oscillometric wrist device accuracy can indeed be affected by wrist anatomy and position (with reference to the heart level), as well as by the wrist cuff characteristics (soft or preshaped). The preshaped cuffs are easier for patients to use but they conform less well than the soft ones to the wrist. Measurement with wrist devices is heavily influenced not only by the level at which the wrist is held but also by its flexion or hyperextension. Furthermore, wrist devices are inherently less accurate because of the difficulties in producing an accurate algorithm to estimate SBP and DBP, as there are two arteries contributing to the oscillometric signal at this site. Wrist devices are, therefore, not generally recommended, because of their inferior accuracy as compared with upper arm devices and because of issues with their correct use according to instructions (www.stridebp.org/bp-monitors). However, their use may be considered in certain specific populations, such as obese or elderly individuals, in whom HBPM using the upper arm is more difficult to perform [80], or in case of novel HBPM devices allowing for night-time automated BP measurements, given that a wrist cuff inflation is likely to produce less interference with sleep quality than an arm cuff inflation [81].

**Arm cuff-based devices** have been shown to be the most reliable both in clinical practice and research, and therefore, their use, coupled with a properly sized cuff, is generally recommended for HBPM.

##### Wearable devices: new perspective for HBPM (Box 8)

The recent technological advances have stimulated the development of wearable systems for health monitoring [82–86]. Before regular adoption of wearable technologies for HBPM, important issues should be addressed, related to system accuracy, efficiency, reliability, legislation, interoperability, services, reimbursement and costs and ethical issues. Preliminary results are promising but there is a strong need for larger, long-term and well designed clinical studies to make these novel solutions really applicable in real-life patients’ care [82–86]. The recent introduction of wearable devices measuring BP at wrist level with the oscillometric method also requires further investigations, also aimed at providing reference values for BP self-measured ‘on the move’ [85].

Box 8 **Selection of devices for HBPM**

**Selection of devices for HBPM**

- Only clinically validated upper arm-cuff devices recommended.
- The devices should be used with appropriate cuff size and according to the manufacturer's instructions.
- Auscultatory devices not recommended except under specific circumstances (e.g. selected cases of arrhythmia).
- Wrist cuff devices not recommended; consider in selected cases when arm-cuff BP measurement is not possible or reliable.
- Finger cuff devices not recommended.
- The clinical usefulness of wearable devices still needs to be established.
- Warn patients that monitors more than 4 years old more likely to be inaccurate [13].
- Validated upper arm cuff devices with personal computer or internet link connectivity and with **s**oftware allowing automatic storage and automatic averaging of 7 days with trend analysis should be preferred.

#### 7.1.2 Clinical validation (Box 9)

As with all BP measurement methods, the use of accurate devices is fundamental for the reliable evaluation of HBP [87]. Aiming to standardize the validation procedures of BP monitors and establish minimum accuracy standards, in the last three decades, the US Association for the Advancement of Medical Instrumentation (AAMI), the British Hypertension Society, the European Society of Hypertension (ESH) Working Group on BP Monitoring and the International Organization for Standardization (ISO), have developed protocols for clinical validation of BP-measuring devices [88]. Unfortunately, most of the devices available on the market have not been subjected to independent validation using one of these protocols [69,87–89]. In 2018, the AAMI, ESH and ISO developed a single universally acceptable standard (AAMI/ESH/ISO, ISO 81060-2:2018), which is intended to replace all previous protocols [90]. Until data on the accuracy of BP monitors using the Universal Standard become plentiful, it is recommended to use only those that have been validated by any of the above-mentioned protocols [69,89]. A device that has been successfully validated in a general population sample might not be accurate in a special population [children, pregnancy, atrial fibrillation, chronic kidney disease (CKD), arm circumference >42 cm] and separate validation in each of these populations is recommended [70,90]. Devices suggested by manufacturers to have equivalent BP measurement function need their equivalence to be independently checked. Updated lists of successfully validated BP measuring devices using an established protocol, and lists of equivalent devices are provided at www.stridebp.org[91].

Box 9 **Validation of HBPM devices**

- Before clinical use, any HBPM device should undergo a clinical validation for accuracy, by means of an established validation protocol.
- In 2018, the AAMI, ESH, and ISO developed a single Universal Standard (AAMI/ESH/ISO, ISO 81060-2:2018), which is intended to replace all previous protocols.
- Updated lists of successfully validated BP measuring devices using an established protocol are provided at www.stridebp.org.
- Devices with BP measurement equivalence to be checked as well as identical devices with different model name in different countries.

#### 7.1.3 Assessing individual device accuracy and the need of device calibration, maintenance or replacement (Box 10)

For yet unexplained reasons, validated oscillometric manometers might sometimes not be accurate in some individuals from the general population. However, there is still no agreement on the need to routinely test device accuracy against a mercury sphygmomanometer or an electronic monitor with screen BP countdown in individual patients, when the device is used for the first time. This is an important issue to clarify, given that it is not possible to identify failure of the oscillometric method in individual patients based on clinical features only [70,92–95].

##### Device calibration and maintenance

Electronic pressure transducers, which represent the heart of an oscillometric BP measuring device, are characterized by a high stability, and generally maintain their accuracy over the years. Thus, it is unlikely that electronic devices might be affected by errors because of loss of calibration [96]. In other words, when a validated automated device yields a BP measurement, the latter should be accurate. Therefore, the finding of persistently abnormal or highly variable readings, without any evident reason (e.g. inappropriate measurement conditions, arrhythmias), might be a sign of device malfunction and should indicate the need for device replacement. Although for aneroid sphygmomanometers used with the auscultatory technique, device calibration is recommended every 6 months [97], in the case of oscillometric devices, regular calibration over time is not generally recommended. However, other BP monitoring device components in addition to the electronic transducer, such as tubing, connections and cuffs, may deteriorate (air leaks, etc.), and may affect accuracy, which emphasizes the need of regular maintenance. Therefore, users should be advised to follow the manufacturers’ recommendations for device maintenance [4]. Finally, given that connected HBPM monitors are becoming more popular and that some of these connected devices allow updates of their software, including the BP measurement algorithm, remotely, special attention should be applied to verify the maintenance of their accuracy.

Box 10 **Device calibration, maintenance and replacement**

- Electronic pressure transducers are characterized by very high stability, and generally maintain their accuracy over the course of many years without the need for calibration.
- Device tubing, connections and cuffs may deteriorate over time, thus also affecting accuracy, and device maintenance is needed in such cases.
- Persistent finding of systematically inaccurate (e.g. highly variable) readings should indicate need of maintenance or replacement.

#### 7.1.4 Cuffs (Box 11)

The size of a cuff bladder is an important component of a cuff-based BP measuring device, which considerably affects its measurement accuracy. Current recommendations for manual auscultatory devices require a cuff with the length of the inflatable bladder covering 75–100% of the mid-arm circumference of the individual and the width covering 37–50% of the length of the mid-arm [90,98]. Using a small bladder (undercuffing) is common in obese adults and leads to overestimation of BP, whereas using a larger cuff (overcuffing) is common in children and leads to BP underestimation [4,90,98,99]. However, there is some inconsistency in the recommendations by scientific societies on the issue of miscuffing [1,4,90,98–100]. For automated oscillometric upper arm-cuff devices, the cuff is also the signal sensor and each cuff size should be validated in an adequate number of individuals according to the validation standard used [90,98]. The above-mentioned rules for the dimensions of the inflatable bladder do not necessarily apply for oscillometric devices, and the accuracy of each oscillometric device should be assessed in association with the recommended cuff size in validation studies [90,98]. The use of wide-range cuffs with oscillometric devices is particularly useful, yet such devices require documentation of their BP measurement accuracy throughout the entire range of arm size of recommended use according to the validation protocol requirements [98]. In individuals with very large arm size (arm circumference >42 cm) the shape of the cuff is also important as the large arm shape is troncoconical [101,102]. Thus, a rectangular (cylindrical) shape cuff is unsuitable as it cannot evenly compress the upper arm and a troncoconical cuff shape seems to be more appropriate [101,102]. However, there is still uncertainty on how to validate devices in people with a large arm size (i.e. >42 cm), as there are issues with the cuff size and shape of the reference (auscultatory) BP measurement as well [102].

Box 11 **Cuffs for HBPM devices**

- The cuff is an important component of a cuff-based BP measuring device, which considerably affects its measurement accuracy.
- Oscillometric devices should be used with the appropriate cuff size according to the individuals’ arm circumference, as instructed by the device manufacturer.
- Wide-range cuffs with oscillometric devices are particularly useful but need validation.
- In individuals with very large arm size, the shape of the cuff is also important. In these individuals, a troncoconical cuff shape is recommended.

### 7.2 Cuffless devices (Box 12) (more details in the online supplemental file S7.2)

Measurement of arterial BP by the brachial cuff sphygmomanometer is still the cornerstone of modern medicine, and this approach has not been yet surpassed by any other noninvasive technology.

However, advances in sensor technology for arterial pulse waveform and speed detection have paved the way for the potential development of devices for cuffless measurement of BP, in the perspective of continuous, beat-by-beat monitoring [103–106] (see online only Supplemental material S7.2). It should be mentioned, however, that established validation standards have not yet been developed to specifically assess the accuracy of cuffless devices and a new ISO standard for such devices is currently under development. Consequently, some of such devices have been validated according to ad hoc draft protocols whereas others according to standard protocols [107,108], thus resulting in heterogenous and difficult to interpret evidence on their accuracy. Therefore, although the cuffless devices are very promising, at present, their use for HBPM is not recommended.

Box 12 **Cuffless devices**

- Measurement of BP by the brachial cuff sphygmomanometer is still the cornerstone of modern medicine.
- Progress in sensor technology for arterial pulse waveform and speed detection has stimulated development of devices for cuffless beat-by-beat measurement of BP.
- Established validation standards have not yet been finalized to specifically assess cuffless devices accuracy, which remains a pending issue for research.

### 7.3 Telemonitoring of home BP (HBPT) values

Despite the demonstrated benefits of HBPM, critical aspects for a proper application of this approach in clinical practice still include data reporting by patients, as well as their transmission to and interpretation by practicing physicians. In general, BP values obtained by patients at home are reported in handwritten logbooks, which are often incomplete and inaccurate (misreporting), and/or illegible, making interpretation of HBPM values difficult. This may discourage physicians from relying on HBPM data for making clinical decisions. A possible solution to this problem is the introduction of HBPM devices equipped with automated memory. However, also in this case, the problems of reporting may persist as data may be stored over different time periods in different devices, making their availability to physicians difficult. In addition, BP measurements taken from different family members might be stored in the same device memory log, thus further increasing the difficulty of their use for hypertension management. A potentially better solution has been provided more recently by progress in information and communication technologies, which in the last decades have made possible the remote transmission of BP values, measured at home, to the doctor's office or hospital, by means of telehealth applications. The conventional approach to home BP telemonitoring is based on computer-tailored data collection and interventions through the Internet mediated by professional service providers, while more modern solutions are based on mobile health technologies using smartphones and their dedicated applications.

#### 7.3.1 Clinical value of HBPT

Implementation of HBPT has the potential to induce an increased patients’ adherence to treatment through their education and involvement in the management of their own health, and to improve doctor--patients relationship. This may help to avoid unnecessary office visits [109–111], and to achieve more satisfactory hypertension control rates [57,112–115], thus improving cardiovascular prognosis [52,116]. Preliminary reports also suggest a possible usefulness of HBPT for self-titration of antihypertensive medication by patients [117], and for comparing antihypertensive treatments in clinical trials [118]. The potential importance of HBPT has been further emphasized by the difficulties in managing patients, including those with hypertension, at the time of coronavirus disease 2019 (COVID-19) outbreak [119]. The main disadvantage of conventional HBPT is the high cost of purchasing and maintaining the system, partly counterbalanced by a reduction in the costs of patients’ management compared with usual care. Advantages and current barriers and limitations to adoption of HBPT are summarized in Box 13.

Box 13 **Advantages and disadvantages of home blood pressure telemonitoring (HBPT). Modified with permission from Omboni *et al.*[120]**

**Advantages of HBPT for patients**

- Improved patient's adherence to treatment and BP control when combined with education and counselling
- Reduced number of office visits and with possible implications for costs of management
- Optimization of therapy facilitating patient--doctor interaction and individual titration

**Advantages of HBPT for doctors**

- Teletransmission of BP readings with possible feedback to ensure doctor's quick update on patient's health status and strict patient monitoring
- Centralized automatic analysis (no need of local software or specific computer skills)
- Promotion of counselling between healthcare operators

**Current Barriers to adoption of HBPT**

- Use of BP telemonitoring out of a clinical research setting in daily practice is difficult to implement as its costs are not yet reimbursed
- Need of adequate infrastructure (mobile network, Internet, connected homes)
- Need for simple and user-friendly devices, possibly integrated in mobile phones, tablets or home appliances
- Need to ensure data security and privacy
- Need of cost-effective systems (full demonstration lacking)

#### 7.3.2 New approaches to HBPM telemonitoring: mobile health: current evidence, future perspectives (Box 14) (more details in the online supplemental file S7.3.2)

In the era of mobile revolution, the widespread use of smartphone technologies, along with the development of smartphones applications for HBPM and remote transmission (T), have opened new perspectives for HBPT (mHealth) [121,122]. Preliminary data from clinical studies and a recent meta-analysis have suggested the value of these technologies in improving patients’ adherence to antihypertensive treatment, and in achieving higher BP control rates [123,124]. In a recent prospective pilot study in patients with treated hypertension, using a telemedicine healthcare management system, which allowed continuous communication between physician and patient via a smartphone application, improved HBP control was achieved in the patients with poor control at baseline [125]. Despite the promising results and future perspectives of mHealth-related interventions [126], there are still some issues in the digital health-based approach that should be addressed before recommending it for widespread clinical use [121].

Box 14 **Advantages and limitations of mHealth technologies**

**Advantages of mHealth technologies**

- Cost-effectiveness
- Accessibility (large proportion of the population owns a smartphone)
- Patients’ empowerment/increased compliance
- Improved achievement of BP control, which might reduce cardiovascular risk
- Devices may be linkable to wearable sensors
- Some devices allow multiparametric recording
- Education and promotion of lifestyle changes
- Possibility of supporting of self-management
- Possibility of recordings during daily activities

**Limits of mHealth technologies**

- Poorly standardized
- Nonvalidated/inaccurate devices and/or m-App are frequently employed
- Demonstration of efficacy through RCT needed
- Privacy and data security are critical
- Though mobile devices are relatively cheap, dedicated software or infrastructures may still be expensive
- Physicians, nurses or technicians may need specific training
- Social, cultural and educational barriers with technology (older people may be less comfortable with technology)
- Nonautomated recording and transmission of BP values might be prone to bias
- Cuff-based devices are still needed (cuffless devices are still not accurate enough)

## 8. CLINICAL APPLICATION OF HBPM IN HYPERTENSION MANAGEMENT

### 8.1 Optimal monitoring schedule (Box 15) (more details in the online supplemental file S8.1)

The selection of the optimal HBPM schedule is based on cross-sectional data examining the reproducibility of average HBP and on outcome data showing its prognostic ability. Cross-sectional studies have mainly focused on the effect of different numbers of readings and days on average HBP, HBP variability and reproducibility; cross-classification according to office and home BP (normotension, white-coat, masked and sustained hypertension); association with ABPM values and association with indices of preclinical target-organ damage [17,18,127–133]. In the International Database on Home blood pressure in relation to Cardiovascular Outcomes (IDHOCO) database (*n* = 4802), the consistency in diagnosing hypertension phenotypes between consecutive monitoring days was improved by averaging more HBP measurements with near perfect agreement after the sixth monitoring day for both office and home BP cross-classification [131]. Furthermore, an increasing number of HBPM days resulted in stronger associations with ABPM, LVMI and urinary albumin, with most of this improvement occurring within the first 4 days (16 readings) [132]. There was no evidence of an improvement when measurements of the first day were discarded [132]. Moreover, there were no differences between morning and evening HBP in their association with indices of target-organ damage [132]. A Finnish study showed that HBP was lower on the weekend than on workdays [134], which might be important in calculating average HBP particularly when the minimum requirement of 3 days is obtained.[135]. Long-term outcome studies provide the most relevant evidence, as they investigate the effect of different HBPM schedules on the prognostic ability of the method, which is the ultimate clinical criterion. Outcome studies in Japan, Greece and Finland with somewhat different HBPM protocols, as well as of the IDHOCO database indicated that the prognostic value of HBPM for cardiovascular disease is increased within the range of 1–7 days, with most of this benefit achieved in the first 3–4 days [131,136–138]. The recommended 7-day schedule should be performed before each office visit, in the commencement phase, the treatment-titration phase, the long-term follow-up phase and whenever there seems to be an unusual change in the BP level (rise or decrease).

Box 15 **Optimal HBPM schedule**

**Timing and duration**

- Before each office visit and whenever an unusual BP change is suspected
- Seven-day monitoring (not fewer than 3 days)
- Routine work days preferred, especially if few days

**BP measurements**

- Duplicate morning and evening measurements
- After a 5 min sitting rest and 1 min between measurements
- Before drug intake if treated

**Interpretation**

- Calculate the average of all measurements (the need of discarding the first day is matter of debate)

**Long-term monitoring of treated hypertension**

- Once or twice per week or month, according to the individual's health status and preference. In case of controlled hypertension: 7 days before each clinic visit, at least over 1 week within 3 months
- Too frequent monitoring (e.g. every day) to be discouraged
- Self-adjustment of drug dosage based on self-measurements to be avoided, if not under guidance by the physician in charge

### 8.2 Diagnostic thresholds (Box 16)

In the 2008 ESH HBPM recommendations, a threshold for hypertension diagnosis with systolic/diastolic HBP of 135/85 mmHg was proposed [4], based on the review of evidence including two meta-analyses [139,140]. Both the analysis of a statistical correspondence to the 140/90 mmHg OBP threshold and the comparison of associated risk of adverse outcomes yielded similar results. Additional evidence regarding the outcome-based thresholds for hypertension diagnosis comes from the IDHOCO database [38]. After analysing only the untreated part of the sample, the proposed approximate HBP thresholds for prehypertension stages 1 and 2 and hypertension stages 1 and 2 amounted to 120/75, 125/80, 130/85 and 145/90 mmHg, respectively. In the IDHOCO database, an OBP value of 140/90 mmHg was a significant predictor of increased risk for all outcomes. The corresponding HBP values were 131.9/82.4 mmHg for cardiovascular events, 132.4/82.8 mmHg for stroke and 131.7/81.2 mmHg for cardiac events [38]. These threshold values did not differ significantly between genders or between age groups below or above 60 years [141]. Conversely, in untreated individuals aged older than 80 years, a significant increase in cardiovascular risk was observed for systolic HBP at least 152.4 mmHg and no risk increase was associated with increased diastolic HBP (the risk level was highest for lowest DBP levels) [142]. Notwithstanding these results, the 135/85 mmHg threshold, already accepted in 2013 European hypertension guidelines [143] was maintained also in the 2018 ESC/ESH guidelines [1]. However, the 2017 US ACC/AHA guidelines recommended to consider this threshold as equivalent to stage 2 hypertension (i.e. ≥140/90 mmHg) [2]. The latter document, by lowering the OBP threshold for hypertension diagnosis (grade 1) definition to 130/80 mmHg raised important questions regarding the corresponding HBP levels to be used for diagnosis [2]. Several studies found that at lower OBP levels the difference with corresponding HBP values becomes much lower [38,144]. ACC/AHA guidelines proposed that the HBP level to identify grade 1 hypertension is the same as for OBP, that is, 130/80 mmHg [38,145–149]. Of note, such lower threshold has a substantial impact on the relative prevalence of sustained, masked and white-coat hypertension [150]. Although in the Ohasama study, changing the thresholds did not have relevant impact on the relationship of these categories with outcome [150], in another study in primary care setting, when 130/80 mmHg threshold was applied, white-coat hypertension cases exhibited some difference in risk compared with normotensive patients (OR 2.0, 95% CI 0.5–7.7) [151].

Box 16 **HBPM thresholds**

- HBP threshold for hypertension diagnosis is at least 135/85 mmHg (systolic/diastolic), which corresponds to OBP threshold at least 140/90 mmHg
- The lower HBP hypertension threshold values (≥130/80 mmHg) proposed by the ACC/AHA guidelines, which are related to the lower proposed and still debated OBP thresholds for hypertension (≥130/80 mmHg), are only partly outcome-based and mostly based on observational surveys
- The suggestion of using lower HBP thresholds for hypertension diagnosis needs to be confirmed by outcome data
- In the very elderly, the BP-related risk increase may begin at higher HBP levels but more evidence is needed

### 8.3 Therapeutic targets and treatment titration (more details in the online supplemental file S8.3)

#### Therapeutic targets (Box 17)

BP targets to be achieved with antihypertensive treatment are generally a controversial issue. In recent United States and European guidelines, new and lower OBP targets were proposed based on the evidence from observational studies, from few specifically designed interventional trials, in particular SPRINT [152], and from recent meta-analyses [153,154]. No direct guidance was provided on the targets for ambulatory or home BP; however, because of inadequate evidence [1,2]. In the HOMED-BP study, participants were randomized to more (<125/<80 mmHg) or less stringent HBP control (125–134/80–84 mmHg). No differences between the groups in terms of cardiovascular events were observed. It should be noted that the achieved BP was very similar in both goups [155]. More recently, the HONEST study provided evidence that achieved systolic HBP above 145 mmHg was associated with significantly higher risk than in a reference group targeting less than 125 mmHg HBP. The risk of this higher HBP category corresponded to that of OBP greater than 150 mmHg. Spline regression analysis suggested some further (although minor) benefit down to HBP of 125 mmHg [156]. Indirect evidence supporting a HBP target less than 135/85 mmHg comes from studies on masked uncontrolled hypertension, in which treated individuals with controlled OBP but HBP higher than the above-mentioned target value had clearly elevated risk compared wiht those with better controlled HBP. In summary, given that the most recent ESC/ESH Hypertension guidelines recommend as a general target for OBP SBP/DBP values 130/80 mmHg or less in treated patients, in the absence of specific evidence on corresponding home BP targets, we might provisionally suggest that HBP values ≤ 130/80 mmHg should also be achieved.

Box 17 **HBPM therapeutic targets**

- Antihypertensive treatment should aim to achieve systolic HBP between 125 and 130 mmHg for most individuals.
- Diastolic HBP targets are less well defined but values 80 mmHg or less might represent a reasonable goal.
- All these suggestions, however, need to be verified in the context of randomized intervention trials with CVD and mortality outcomes.

#### Treatment titration (Box 18)

Although titration of antihypertensive treatment is a crucial part of the management of patients with high BP [157], titration on the basis of few OBP measurements in primary care may be suboptimal. HBPM offers the unique possibility to evaluate BP on treatment and titrate BP medications either by professionals or by patients themselves (if educated) based on a higher number of readings. In the past years, several studies have assessed the effectiveness of different titration strategies guided by HBPM. The THOP and the HOMERUS trials were the first to examine the efficacy of antihypertensive titration using HBPM [158,159], but their results are undermined by major limitations as the same BP target for both randomization arms was considered, and differences in BP titration and blinding were maintained with the prescribing physician unaware of randomization group. In 2014, a study, which randomized patients with untreated hypertension to management based on clinic and ABPM measurement or HBPM alone showed that after 1 year, there was no significant difference between groups in LVMI regression. [160,161] Another study explored whether a telemonitoring-based intervention [162] provided a better result than usual care. The results showed a significantly better reduction in BP in the tele-HBPM group compared with controls, albeit with relatively short follow-up.

Most recently, the TASMINH4 study aimed to assess both the longer term (12 month) effect of titration using HBPM and the influence of telemonitoring over and above HBPM with simple paper-based feedback on hypertension control. After 12 months, both HBPM groups had significantly lower SBP than those titrated based on clinic readings and the telemonitoring group also had lower BP at 6 months suggesting quicker titration with telemonitoring [52,163]. The TASMIN-SR trial [55] developed the concept of self-management guided by primary care physicians based on the results of self-monitoring. The intervention was compared with usual care in higher risk patients with hypertension and after 12 months, the mean BP had decreased to 128.2/73.8 mmHg in the intervention group and to 137.8/76.3 mmHg in the control group, with a difference of 9.2/3.4 mmHg after correction for baseline BP. Despite the clear evidence that HBPM is effective in reducing BP, as confirmed by a recent meta-analysis [164], cost effective and well tolerated by patients, there are still some open questions, such as how to integrate home self-BP monitoring with clinical records and, most of all, how to choose patients for self-titration, an issue currently under investigation [165].

Box 18 **HBPM and treatment titration**

- Physicians using HBPM to titrate antihypertensive medication can achieve better hypertension control than with OBP alone
- HBPM and guided self-titration lead to significantly lower BP than titration guided by clinic readings.
- There are still some open questions, such as how to integrate HBPM with clinical records and which patients to select for self titration.

### 8.4 HOME vs. ABPM (more details in the online supplemental file S8.4)

When combined with OBP, both HBPM and ABPM can identify white-coat and masked hypertension in untreated and treated individuals. The threshold for BP normality is indicated as similar for HBP and ambulatory daytime BP (≥135/85 mmHg), whereas a lower threshold is recommended for 24-h ABP (≥130/80 mmHg) [1,2,9,166]. The similar diagnostic ability of HBPM and ABPM is probably because of the fact that both methods provide multiple measurements taken away from the office setting in the usual environment of each individual. However, there are also important methodological differences between them, as HBP is measured only after few minutes rest, in a standardized sitting posture at home and during the day, whereas ABP is measured at different postures (sitting, standing and lying), in different environments (work, home, other) and during routine daytime activities and night-time sleep. Thus, HBPM and ABPM are similar but are not identical methods and the diagnostic agreement between them is sometimes a challenging clinical issue [20]. ABPM has been indicated as the most reliable and accurate measurement of BP for diagnosing hypertension and while assessing the response to therapy [2], as it provides information about specific patterns of BP behavior, such as nocturnal dipping, morning surge and short-term variability over 24-h [2,166–168]. At variance from ABPM, HBPM it is widely available, relatively inexpensive and well accepted by patients, particularly for repeated monitoring. Consequently, current guidelines recommend also HBPM as a method for the evaluation of BP in untreated individuals with suspected hypertension and, more so, for monitoring the long-term BP control in treated hypertensive patients [4,169]. An important issue to consider is the reproducibility of BP information provided by HBPM and ABPM, respectively, which was shown to be similar by a direct comparison of HBPM and ABPM data in the same individuals [18,170], or even higher for HBPM [20]. In a recent meta-analysis of 58 studies, diagnostic performance of HBPM was slightly higher than OBP. Indeed, a normal OBP can be accompanied by elevated HBP in case of masked hypertension. However, some individuals with normal HBP showed elevated BP from 24-h ABPM, suggesting that ABPM is still necessary for confirming the diagnosis of hypertension [171]. A recent study, in agreement with previously published PAMELA study data [172] indicated that individuals showing a diagnostic disagreement between their home and ambulatory BP may have cardiovascular risks that are intermediate between those with sustained home and ambulatory normotension and hypertension [173]. However, no precise indications are given in available guidelines on when and in which particular patients to use ABPM or HBPM and the decision to use ABPM or HBPM often reflects the preference of the individual patient and of the healthcare provider. The healthcare system in which a patient is managed may also be a factor. Although it remains unclear whether one approach is superior to the other for diagnosing hypertension and monitoring BP control, it has to be acknowledged that HBPM and ABPM are equivalent but they measure different aspects of the BP behaviour, so they represent complementary rather than alternative approaches. See Box 19. The 2018 ESC/ESH Hypertension guidelines emphasize the complementary role of HBPM and ABPM and recommend that both methods should be used, whenever possible [1]. Unfortunately, this is not often possible in low-resource settings because of healthcare costs and unavailability of these approaches, in particular ABPM, in daily practice, which might make HBPM the preferred out-of-office BPM method [10,11].

Box 19 **When to use ABPM or HBPM**

**When to use ABPM or HBPM**

- ABPM: for initial diagnosis, best if accompanied by 7-day HBPM
- HBPM: for long-term follow-up unless special situations need to be explored (i.e. nocturnal hypertension in obstructive sleep apnea patients, job strain, increased short-term BPV, morning BP surge, etc.)
- HBPM should be used whenever ABPM is not available or not tolerated
- Disagreement between ABPM and HBPM may occur. In such a case both HBP and ABP values should be considered, on the background of standardization and reproducibility issues of each method

#### HBPM vs. ABPM: clinical relevance (Box 20)

**Advantages and disadvantages of HBPM and ABPM (**Table 2**)**

**TABLE 2:** Comparison of Advantages and disadvantages of HBPM vs. ABPM

ABPM	HBPM
Advantages Can identify white-coat and masked hypertension Stronger prognostic evidence Night-time readings Measurement in real-life settings Additional prognostic BP phenotypes Abundant information from a single measurement session, including short-term BP variability Disadvantages Expensive and sometimes limited availability Can be uncomfortable, particularly at night Cannot be repeated too frequently	Advantages Can identify white-coat and masked hypertension Cheap and widely available Patient engagement in BP evaluation, which improves compliance with treatment and BP control Easily repeated and used over longer periods to assess day-to-day BP variability Preferred to ABPM by most patients, particularly for repeated use Disadvantages Only BP at home and at rest is evaluated Potential for measurement and reporting errors Many HBPM devices on the market have not been validated No nocturnal readings (with most devices) HBPM may lead to excessive anxiety about BP levels

ABPM, ambulatory blood pressure monitoring; HBPM, home blood pressure monitoring.

Box 20 **Clinical use of ABPM and HBPM: when and how**

- Diagnosis of hypertension should be made combining office with out-of-office BP monitoring
- HBPM and 24-h ABPM show similar reproducibility and prognostic ability, and can both be considered, depending on availability, preference and tolerability
- HBPM does not give the same information on BP behaviour as ABPM does, but it is widely available, relatively inexpensive and well accepted by patients particularly for repeated long-term use, as it causes less discomfort and restriction of daily activities and sleep than ABPM
- HBPM requires reliable recording of self-measured BP. Too frequent HBP measurements should be avoided to prevent anxiety about individual's BP levels
- ABPM and HBPM are complementary and not interchangeable techniques, providing complementary information on BP in different conditions and over different time periods
- HBPM, in particular when used with co-interventions, may increase patient's adherence to treatment, awareness and active involvement, leading to improved BP control rates
- How to choose HBPM or ABPM: individualized approach based on patient's characteristics (BP values, cardiovascular risk, target organ damage) and availability of HBPM and ABPM. In case of disagreement between methods, both HBP and ABP data should be considered, keeping in mind that ABPM has larger amount of outcome data available

## 9. SPECIAL CONDITIONS

### 9.1 HBPM in children and adolescents (Box 21)

Considerable emphasis on the methodology of OBP measurements has been put in the recent European and American guidelines for pediatric hypertension, by stressing the importance of standardized conditions, use of validated monitors with appropriate cuff size and performing multiple measurements [174,175]. ABPM is currently regarded as the reference method for hypertension diagnosis in children and adolescents as white-coat, masked and nocturnal hypertension are as common as in the adults [174–178]. The role of HBPM in the evaluation of pediatric hypertension remains largely unrecognized and inadequately investigated, even though the method is being used in clinical practice in children with hypertension [176,177]. HBPM appears to have several potential advantages over both OBP and ABPM, including user convenience and acceptance, and the ability to obtain multiple measurements in the usual environment over several days, weeks or months [174–179]. Preliminary evidence for HBPM in children shows that, as in the adults: its reproducibility is superior to that of OBP measurements and close to that of ambulatory monitoring [21,176–178]; there is close agreement between home and ambulatory BP monitoring in diagnosing hypertension phenotypes within the range of 80–85% [176–179]; the association of HBPM with several indices of preclinical target-organ damage, mainly LVMI, appears to be superior to OBP measurements and similar to ABPM [176–178,180,181]. An automated oscillometric device, which has been successfully validated in adults may not be accurate in children [90,91,182]. Thus, HBPM in children must only be performed using automated upper arm devices that have been validated specifically in this population. Unfortunately, very few oscillometric home BP monitors have been tested in children [91,182]. Lists of electronic BP monitors successfully validated in children are available at www.stridebp.org[91]. The normalcy range of HBP has been investigated in a single cross-sectional school-based study in 778 children and adolescents, which provided percentile tables according to gender and height (Table 3) [183]. Home BP at the 50th centile for gender and height represents the ‘usual’ HBP level, whereas HBP equal or higher than the 95th percentile represents the threshold for home hypertension [183]. A recent study investigating the HBP normalcy in adolescents in Brazil showed no clear differences in distribution patterns of the 95th percentiles for HBP in European and non-European adolescents [184]. It should be mentioned that, in contrast to the adults in whom home and daytime ambulatory BP have similar levels, in children and adolescents, HBP is considerably lower than daytime ABP, which is attributed to the higher level of physical activity of the young individuals during the day [176,185]. Preliminary evidence on the optimal HBPM schedule in children showed that 6-day monitoring (no less than 3 days) with duplicate morning and evening measurements taken after few minutes rest in the sitting position is adequate, which is in line with the evidence in the adults [186]. In young children, measurements should be taken by parents, whereas in the adolescents, self-measurement is usually appropriate. Practical recommendations for HBPM in children are shown in Box 21.

**TABLE 3:** Normalcy table for home blood pressure in children and adolescents by gender and height

		Percentiles for boys (*n* = 347)			Percentiles for girls (*n* = 420)	
Height (cm)	*N*	50th	95th	*N*	50th	95th
120–129	23	105/64	119/76	36	101/64	119/74
130–139	51	108/64	121/77	51	103/64	120/76
140–149	39	110/65	125/77	61	105/65	122/77
150–159	41	112/65	126/78	71	108/66	123/77
160–169	45	115/65	128/78	148	110/66	124/78
170–179	91	117/66	132/78	46	112/66	125/79
180–189	57	121/67	134/79	7	114/67	128/80

The values are SBP/DBP measures expressed in mmHg. Reproduced with permission from [183].

Box 21 **Instructions for HBPM in children and adolescents. Modified with permission from [177]**

**Devices**

- Automated electronic (oscillometric) upper arm specifically validated in children
- Appropriate cuff size to fit the individual's arm circumference
- Automated storage and averaging of readings, or mobile Bluetooth connection, or PC link, or tele-monitoring for unbiased reporting

**Conditions**

- Measurements in a quiet room after 5 min sitting rest
- Back supported, arm resting at heart level, feet flat on floor
- Avoid talking during and between measurements
- Measurements by parents in young children, or self-measurements in adolescents

**Schedule**

- Home monitoring for seven routine school days (no less than 3 days)
- Duplicate morning and evening measurements on each day with 1 min intervals

**Interpretation**

- Calculate the average of all measurements after discarding the first day
- Evaluate the average value using the normalcy data for HBP in children
- Average HBP at least 95th percentile for gender and height indicates home hypertension

### 9.2 HBPM in pregnancy (Box 22)

Hypertensive disorders of pregnancy, including preeclampsia, complicate up to 10% of pregnancies worldwide, being one of the major causes of maternal and perinatal morbidity and mortality worldwide [178,187–188]. BP in pregnant women should be measured at every antenatal visit. However, even antenatal schedules may not be sufficiently frequent to identify fulminant preeclampsia where onset and progress can be rapid and often asymptomatic [178]. In addition, white-coat hypertension is common in pregnancy, especially towards its end (15–35%) [14,188]. Both home and ambulatory BP monitoring have been shown to more accurately characterize BP in pregnancy as in nonpregnant individuals [178]. HBPM is well accepted by pregnant women, and results in fewer antenatal visits overall, while improving surveillance [189]. Two large trials are currently exploring the place of self-monitoring in pregnancy [190]. Analysis of individual patient data from self-home monitored and clinic BP readings from eight studies (*N* = 758) did not reveal any systematic difference between these two methods, suggesting that appropriate treatment and diagnostic thresholds for self-HBPM during pregnancy would be equivalent to standard clinic thresholds [14]. However, average BP values in this analysis were in the range of 117–125 mmHg, which does not represent pregnant women with hypertension. More research is needed to define the threshold of hypertension based on HBP measurements during pregnancy. An automated oscillometric device, which has been successfully validated in adults may not be accurate in pregnancy or preeclampsia [90,191]. Thus, these devices should be specifically validated in normotensive and hypertensive pregnant women and also in preeclampsia [90,191]. Lists of BP monitors, which have been successfully validated in pregnancy including preeclampsia are available at www.stridebp.org[91]. The sitting position appears to be appropriate for HBPM during pregnancy [178].

Box 22 **HBPM in pregnancy**

- HBPM is well accepted by pregnant women, and results in fewer antenatal visits overall, while improving surveillance and allowing diagnosis of white-coat hypertension
- More research is needed to define the threshold of hypertension based on HBP measurements during pregnancy
- HBPM devices should be specifically validated in normotensive and hypertensive pregnant women and also in preeclampsia

### 9.3 People with large arm circumference (Box 23)

Large arm circumference is typical, but not exclusive, of the obese patient, an issue to consider when performing HBPM in these individuals. This observation is relevant given that high BP variability and, most of all, elevated prevalence of white-coat hypertension and masked hypertension among obese patients make HBPM a fundamental tool to appropriately define the BP profile in such patients [192,193]. One of the main issues in the management of patients with large arm circumference is the limited availability of properly sized cuffs, which can end up in an overestimation of BP whilst using a standard size cuff [194,195]. Other issues, which may affect accuracy of HBPM include the conical shape of large arms and the combination of large arm circumference with short humerus length [196] (see also session on cuff size and shape). The use of wrist devices may help avoiding these difficulties and may represent a potential alternative for HBPM in obese individuals whenever upper arm cuff devices cannot measure BP but further investigation to prove this possibility and technological improvement is needed.

Box 23 **HBPM in individuals with large arms**

- Difficulty in performing HBPM in individuals with large arms is because of a number of factors (lack of proper sized cuffs, conical-shaped arms, short humerus length)
- Studies are needed to explore the possible usefulness of wrist devices

### 9.4 Patients with chronic kidney disease (Box 24) (more details in the online supplemental file S9.5)

Patients with CKD often exhibit abnormal 24-h BP profiles, including increased short-term BPV, reduced nocturnal BP dipping and, not infrequently, reverse dipping [197–199]. Thus, defining hypertension control in such patients is challenging, and HBPM has been shown to be superior to OBP in identifying lack of hypertension control [200]. Given that in patients with CKD, adequate BP control reduces not only the decline in kidney function but also cardiovascular morbidity and mortality, an accurate assessment of BP status is a key to the optimal management of patient with reduced kidney function [201,202]. A meta-analysis shows that among patients with CKD, both white-coat hypertension and masked hypertension are common; about 40% of patients thought to have normotension (or adequately treated hypertension) in fact had hypertension at home whereas about 30% of patients thought to have hypertension had normotension at home [203]. HBPM can better define the progression to kidney failure, including end-stage kidney disease (ESKD), and cardiovascular risk among patients with CKD [202]. Therefore, HBPM in CKD patients is important [202]. Patients with CKD on haemodialysis are profoundly different from patients with CKD who are not on dialysis; this is so because of varying states of volume excess among patients and volume accumulation in the interdialytic period [204–206]. In fact, compared with peridialysis BP, HBP has a stronger association with LVH in patients on haemodialysis [207]. Furthermore, considering ABPM as a reference standard, HBP and not predialysis or postdialysis BP, offers the best combination of high sensitivity and high specificity for the diagnosis of hypertension [208]. In contrast to peridialytic BP recordings, high home SBP relates to an increased mortality in dialysis patients [209,210]. It also carries greater prognostic information. Lastly, it should be mentioned that in ESKD patients, the accuracy of the oscillometric devices is reduced because of increased arterial stiffness and presence of an arteriovenous fistula but only a few of them have been successfully validated in these patients [211].

Box 24 **HBPM in CKD patients**

- HBPM has been shown to be superior to OBP in identifying lack of hypertension control
- Masked hypertension is frequent in patients with CKD
- In CKD, HBPM better predicts cardiovascular events, progression to end-stage kidney disease (ESKD) or death than OBP
- HBP is better associated with left ventricular hypertrophy in patients on haemodialysis compared with peridialysis BP
- HBPM twice daily, after the midweek dialysis for 4 days, shows satisfactory agreement with interdialytic 44 h ABPM.
- Separate validation of oscillometric HBPM devices might be considered in ESKD patients
- Given the high prevalence of elevated nocturnal BP levels in CKD, usefulness of HBPM devices with nocturnal BP function should be tested

### 9.5 HBPM in patients with arrhythmia (atrial fibrillation) (Box 25)

HBPM in patients with arrhythmias, especially atrial fibrillation, raises special concerns [212,213]. Particularly in the elderly, hypertension and atrial fibrillation often coexist as their prevalence is rising considerably with increasing age, as clearly demonstrated in recent outcome trials of novel oral anticoagulants where 50–90% of the atrial fibrillation participants were hypertensive patients [214]. In the presence of atrial fibrillation, both the manual auscultatory and the automated oscillometric measurement of BP are difficult and uncertain because of variations in ventricular filling time, stroke volume and contractility, all of which increase beat-to-beat BPV [212]. Thus, issues with the accuracy of automated BP measurement and its clinical relevance in atrial fibrillation are of high importance. To account for the increased BPV in atrial fibrillation, it is recommended that several measurements should be averaged using the auscultatory method, and that the automated oscillometric devices should be avoided as most of them have not been validated for accuracy in such patients [1]. However, self-measurement using the auscultatory method at home is not feasible in the elderly hypertensive patients with atrial fibrillation. The current evidence from published validation studies of automated oscillometric BP monitors in atrial fibrillation is rather limited and methodologically heterogeneous [15,212,215]. However, it appears that there is reasonable accuracy of these devices in measuring SBP in the presence of atrial fibrillation but with a small yet consistent overestimation of DBP (pooled automated minus auscultatory SBP difference 1 mmHg, 95% CI −0.1 to 2.1, and DBP 2.1 mmHg, 95% CI 0.1--4.0) [212]. In such a context, triplicate rather than duplicate measurements should be considered, because of increased beat-to-beat variability [212]. An invasive study in atrial fibrillation patients with high ventricular rate showed that there is larger underestimation of oscillometric SBP compared with intra-aortic measurement [216]. The clinical relevance of BP measurement in atrial fibrillation has been demonstrated in a recent meta-analysis, which showed that both manual auscultatory and automated oscillometric OBP measurements predict stroke or systemic embolism in atrial fibrillation patients, and follow-up BP control has superior predictive ability than baseline BP [217]. Thus, despite their inherent instability, BP measurements in atrial fibrillation are clinically relevant as in individuals with sinus rhythm. These findings, together with the fact that in the elderly, systolic hypertension is far more common and important for prognosis, suggest that automated devices should be used for HBPM even in the presence of atrial fibrillation [212], in particular when ventricular rate is controlled by treatment. HBPM with specific devices has also been suggested as a means to screen for presence of atrial fibrillation. In fact, novel oscillometric HBPM devices have been developed, which are equipped with an algorithm specific for atrial fibrillation detection during routine automated BP measurement. Accumulating evidence suggests that screening for atrial fibrillation in the elderly using an atrial fibrillation-specific algorithm during routine automated office, home or ambulatory BP measurement has high diagnostic accuracy [212,218,219]. Two studies have shown that HBPM with automated atrial fibrillation detector might be useful for early detection of asymptomatic atrial fibrillation in elderly individuals with hypertension [219,220].

Box 25 **HBPM in patients with arrhythmias**

- Particularly in the elderly, hypertension and atrial fibrillation often coexist
- In the presence of atrial fibrillation, both the manual auscultatory and the automated oscillometric measurement of BP are difficult and uncertain because of increased beat-to-beat BP variability
- There are few and methodologically heterogeneous validation studies of automated oscillometric BP monitors in atrial fibrillation. It appears that with atrial fibrillation, there is reasonable accuracy of these devices in measuring SBP but with a small yet consistent overestimation of DBP
- Despite their inherent instability, BP measurements in atrial fibrillation predict outcome as in individuals with sinus rhythm
- Oscillometric HBPM devices equipped with an algorithm specific for atrial fibrillation detection during BP measurement can be useful

## 10. NOCTURNAL HBPM (Box 26) (more details in the online supplemental file S10)

There is evidence that nocturnal BP assessed by ABPM has superior prognostic ability compared with OBP and daytime ambulatory or home BP measurements [221,222]. Recent technological development of electronic home monitors has enabled automated measurement of home BP during night-time sleep [81,223]. These novel HBPM devices are programmed to be initiated by a trigger preasleep measurement by the patient and later take few automated prescheduled measurements during night-time sleep [81,223]. These devices can be used for consecutive nights to obtain a sufficient number of asleep BP readings, thus possibly increasing the reproducibility of night-time BP. A meta-analysis of six studies including 1404 individuals compared nocturnal HBPM with night-time BP by ABPM and showed pooled correlation coefficients between them of 0.70/0.72 and pooled differences of 1.4/−0.2 mmHg (systolic/diastolic) [170]. In the same meta-analysis, 2 studies including 212 individuals investigated the agreement between nocturnal HBPM and ABPM in detecting nondippers and showed weighted agreement of 77%, which is close to the reproducibility of each of the two methods [170]. Moreover, similar pooled correlation coefficients of nocturnal systolic HBP and ABP were reported with LVMI, urinary albumin excretion and cIMT [170]. The Japan Morning Surge-Home Blood Pressure (J-HOP) Nocturnal BP Study in 2545 participants showed night-time systolic HBP to predict incident cardiovascular events, independent of office and morning home BP [224–226]. Regarding the optimal schedule of night-time HBPM, a single study showed that a two-night HBPM schedule with three automated measurements scheduled per night (total of six asleep BP readings) appears to be the minimum requirement for a reliable assessment of night-time HBPM, providing reasonable agreement with nocturnal ABPM and association with indices of preclinical organ damage [227]. These findings are in line with the current recommendations for assessing night-time ambulatory BP with a minimum requirement of seven readings [228]. Furthermore, in a crossover study, the reliability of nocturnal HBPM appeared to be similar between a schedule of measurements at 2, 3 and 4 h after the chosen bedtime and that with measurements at fixed time points (at 0200, 0300 and 0400 h) [229]. Obstructive sleep apnoea is known to be associated with nocturnal hypertension and nondipping profile, which are associated with adverse prognosis. Preliminary evidence suggests that nocturnal HBPM can be used as an alternative to ABPM in the investigation of nocturnal BP in patients with sleep apnoea [230,231]. A novel home BP monitor developed specifically for the evaluation of patients with obstructive sleep apnoea, is able to trigger asleep BP measurement during episodes of hypoxia (reduced oxygen saturation) [231]. In conclusion, there is accumulating evidence that nocturnal HBPM is feasible and appears to be a reliable alternative to ABPM for the evaluation of asleep BP. A recent consensus statement presents a systematic review of the current evidence on nocturnal HBP, and highlights the potential of the method, the remaining research questions and preliminary recommendations for its clinical application [81]. Whether wrist devices may perform better at night than arm cuff devices, because of less pronounced interference with sleep patterns, this is an issue, which deserves to be further explored.

Box 26 **Nocturnal HBPM**

- Technological development of HBPM devices has enabled automated measurement of HBP during night-time sleep
- Available data suggest reasonable correlation and similarity between asleep BP values obtained by HBPM and ABPM
- A two-night HBPM schedule with three automated measurements scheduled per night (total of six asleep BP readings) appears to be the minimum requirement for a reliable assessment of night-time HBPM
- Nocturnal HBPM seems to be feasible also as a reliable alternative to ABPM in the detection of nocturnal hypertension and nondippers and the investigation of patients with sleep apnea, but more research is needed
- Whether wrist devices may perform better at night than arm cuff devices, because of less pronounced interference with sleep patterns, this is an issue, which deserves to be further explored

## 11. ORTHOSTATIC HYPOTENSION (Box 27)

Orthostatic hypotension is a common condition associated with adverse cardiovascular prognosis [232,233]. Screening for orthostatic hypotension consists of BP measurements in supine (or sitting) and standing position during clinical consultations [234]. However, orthostatic hypotension is poorly reproducible; thus assessments carried out at the doctors’ office are likely to underestimate its true prevalence [235]. HBPM can improve orthostatic hypotension diagnosis without compromising the quality of the postural BP assessment, as shown by Cremer *et al.*[236] who demonstrated that in 505 mostly hypertensive patients, orthostatic hypotension prevalence defined as the presence of one episode of orthostatic hypotension detected by HBPM was 37.5%, much higher than orthostatic hypotension prevalence measured in the same cohort in a clinic setting (15%). Orthostatic hypotension is a common finding in elderly patients because of their impaired baroreceptor sensitivity [237], and is often associated with hypertension, dementia, other neurodegenerative diseases (e.g. Parkinson's disease), atrial fibrillation, diabetes and heart failure [238–241]. However, in the elderly individuals, it may be difficult to use HBPM because of physical and/or cognitive dysfunction of the users [242]. The diagnostic accuracy of HBPM in detecting orthostatic hypotension as compared with ABPM needs to be explored. The potential usefulness of wearable devices for self BPM in detecting orthostatic hypotension requires to be investigated.

Box 27 **HBPM in orthostatic hypotension**

- Orthostatic hypotension is common in the elderly but is poorly reproducible. Thus, assessments carried out during consultations may underestimate the true prevalence
- HBPM can improve orthostatic hypotension diagnosis, by allowing for repeated measurements in daily life, also in association with the peak drug effect and associated symptoms
- The diagnostic accuracy of HBPM in detecting orthostatic hypotension as compared with ABPM needs to be explored

## 12. HOME BLOOD PRESSURE VARIABILITY (Box 28) (more details in the online supplemental file S12)

BPV has been assessed for many years by mostly focusing on 24-h ABPM recordings. More recently, evidence has been provided also on the clinical relevance of BPV assessed over longer periods, that is, based on OBP measured in different visits (visit-to-visit BPV, VVV) or on HBP measurements obtained over a week or even longer time intervals (home BPV, HBPV) [243]. As HBPV is easier to obtain than VVV in a standardized manner, it has even been suggested that it might be the ideal approach to assess BPV [244]. Increased day-by-day HBPV has been associated with advanced age, female gender, increased arterial stiffness, elevated mean BP values, low BMI, low heart rate, excessive alcohol intake, cigarette smoking, cardiovascular disease, diabetes, diabetic nephropathy and sedentary lifestyle [245]. Studies focusing on treated hypertensive patients have found a higher day-by-day BPV among these individuals compared with untreated individuals [38,140], also reporting higher values of HBPV in case of treatment with beta-blockers, short duration of treatment [246] and increasing number of antihypertensive drugs [247]. Regarding the optimal methodology of HBPV assessment, two principal elements should be considered: how HBP data are collected; what estimates of HBPV should be considered. Studies addressing the predictive value of HBPV for HMOD are characterized by heterogeneous methodology and by discrepant results [245]. Conversely, the evidence supporting the relationship of HBPV with clinical outcome is more consistent. In the IDHOCO database, all indices of systolic/diastolic HBPV (SD, CV, ARV, VIM) were independently associated with all-cause and cardiovascular mortality [248] although did not significantly improve risk stratification. A meta-analysis of observational cohorts and of clinical trials reported a significantly increased risk of cardiovascular events, cardiovascular and all-cause mortality in relation to an increased HBPV after accounting for confounders [249]. HBPV was superior to OBP variability in the Didima study [250]. Morning day-by-day HBPV may have superior prognostic value as compared with morning--evening or evening HBPV [137,251]. Regarding potential threshold values for mid-term day-by-day BPV, the results of the IDHOCO study indicate that the risk of cardiovascular morbidity and mortality steeply increased in the highest decile of systolic/diastolic HBPV distribution (CV ≥11/12.8%, respectively) [252]. These data need, however, to be validated by other studies. Also, there is no unequivocal evidence that HBPV reduction may provide benefits in terms of HMOD or risk of events independently of average BP reduction, because of lack of ad hoc intervention trials [253,254]. In conclusion, HBPV is an independent outcome predictor and might be particularly useful for long-term monitoring but the available evidence does not support its application in clinical practice.

Box 28 **Home BPV**

- HBPV can be measured by considering readings obtained day-by-day over 1 week of HBPM
- HBPV is related to several possible determinants and has been reported to predict outcome after accounting for confounders. Available evidence, however, comes from heterogeneous studies
- There is no clear evidence that any treatment modality might be superior in reducing HBPV
- HBPV is an independent outcome predictor but the available evidence does not support its application in clinical practice

## 13. BARRIERS FOR CLINICAL USE, COST-EFFECTIVENESS AND PATIENTS’ PREFERENCE (Box 29)

### 13.1 Barriers for clinical use of HBPM (more details in the online supplemental file S13.1)

Adoption of HBPM is still challenging because of barriers that involve three general domains: cultural, structural and financial [255–258]. From a cultural standpoint, poor education on the need for regular BP monitoring still exists among doctors and patients with the former being unable to implement properly local guidelines and the latter being often unaware of the importance of cardiovascular risk factors detection and control. Such cultural barriers are relevant and are often enhanced by the need of more robust evidence on the benefit of HBPM, including additional studies focusing on HBPM impact on prognosis, advantageous cost/benefit ratio and HBP thresholds/targets for treatment. Some more structural barriers not only limit the availability of HBPM and include the lack of adequate infrastructures for HBPM implementation at a population level and for data transfer to the physician in charge but also the need for simpler and more user-friendly devices, with simple functioning and readable displays capable to ensure data security and privacy. Lastly, financial issues represent still a major problem for patients highlighting the need of cost-effective systems made by cheap and integrated devices with the aim of being reimbursed by healthcare services or insurances. In this context, we should consider that diffusion of HBPM in developing countries may be importantly limited by income levels of the population well below what occurs in more developed areas of the world.

### 13.2 Cost-effectiveness of HBPM

Published data on the cost-effectiveness of HBPM have been conflicting [259]. Without other co-interventions, HBPM has been found to provide only a small BP-lowering benefit that is not sustained over time [260,261]. However, HBPM by itself may not improve BP control but can improve therapeutic inertia and thereby provide a substantial benefit [56]. In fact, the BP-lowering benefit of HBPM has been greater when used with co-interventions (e.g. telemonitoring, pharmacist visits) [262]. Therefore, the cost-effectiveness of HBPM needs to be considered within the context of these co-interventions, and in relation to the associated costs. It is important to distinguish HBPM from the broader category of self-measured BP, which may also include the use of kiosks or measurements obtained by a patient using an automated device at their healthcare provider's office [257]. A recent systematic review showed that although the accuracy of both office and home BP for the diagnosis of hypertension has increased, ambulatory BP remains the most cost-effective option to confirm a diagnosis of hypertension, an issue currently under debate [262]. Individual trials of self-BP monitoring at home have also shown cost-effectiveness. In primary care, the TASMINH4 study showed that titration of antihypertensives using self-monitored BP was cost-effective, with similar probabilities of cost-effectiveness from manual and telemonitored transmission of readings [163]. A recent study showed that HBP telemonitoring accompanied with pharmacist management effectively lowered BP levels, with an estimation for a significant reduction in costs for the health system by avoiding cardiovascular events over 5 years [263].

### 13.3 Patients’ preference and healthcare provider concerns

HBPM is preferred to ABPM by the majority of patients as it is less intrusive in their daily life. ABPM has been reported to cause discomfort in 55% of patients and the need of severe restrictions of daily activities was reported by 30% of patients. However, although there is limited data for direct comparison with HBPM [264,265]. This is perhaps more so in minority ethnic populations [266]. Concerning healthcare providers, there is a need of specific education and update on use of contemporary HBPM devices and on the criteria for their selection and use. Pharmacists, who are often in charge of selling the devices, should be informed and constantly updated on the devices that have been successfully validated according to internationally acknowledged validation protocols, as well as on their specific features, including the availability of cuffs of different size, wide-range cuffs or specific algorithm for arrhythmia detection. The should also be informed on which devices have been validated for special populations, such as pregnant women or children and adolescents. Regarding physicians, they should also be educated on how to instruct patients to make proper use of HBPM devices. In particular, indications should be provided on how to prescribe a HBPM and what explanation should be given to patients, also regarding where to buy and how to choose a reliable HBPM device. Finally, physicians concerned about the possible ‘neurotic’ performance of an excessive number of BP measurements by anxious patients should be trained on how to educate and instruct such patients.

Box 29 **HBPM: barriers, cost-effectiveness and patients’ preference**

- Adoption of HBPM is still challenging because of cultural, structural and financial barriers
- Taken together, preference of patients and providers and cost-effectiveness data support the use of HBPM

## 14. HBPM IN CLINICAL RESEARCH (Box 30) (more details in the online supplemental files S6.1 and S14)

During the last two decades, HBPM has been used increasingly in clinical hypertension research [17,155,267–273]. Its multiple advantages lead to superior diagnostic reliability and measurement reproducibility, ensuring improved accuracy of clinical trials as compared with the use of OBP measurements, and thereby leading to smaller study sample size and lower research costs, together with better patients’ acceptance, particularly for longer term trials [17].

**HBPM use may improve selection of study participants** as it offers more accurate definition of hypertension phenotypes and allows identification of individuals with white-coat and masked hypertension.

**HBPM improves evaluation of the duration of action of antihypertensive drugs** as it allows to obtain measurements before drug intake and postdose, thus providing information on the ‘trough’ and ‘plateau’ (not peak) effect calculating the morning-to-evening (M/E) home BP ratio. Moreover, as already mentioned, HBP has no regression to the mean phenomenon during both pretreatment drug-free period and antihypertensive drug monotherapy period [274].

### HBPM also allows for evaluation of drug-induced effects on BPV

HBPM allows tracking specific BP changes during usual activities or interventions (i.e. postprandial hypotension).

Box 30 **HBPM in a research setting: usefulness in clinical trials**

- **General advantages:** availability of multiple readings over time, high reproducibility and diagnostic accuracy, correlation with early HMOD and cardiovascular events, easily acceptable to patients.
- **Improved selection of study participants**
- **Improved study power and reduced sample size**
- **Improved evaluation of the duration of action of antihypertensive drugs**
- **Better support to chronotherapy studies**
- **Evaluation of drug-induced effects on BPV**

## 15. REMAINING UNRESOLVED RESEARCH QUESTIONS

1. Diagnostic agreement between ABPM and HBPM needs further evaluation.
2. More longitudinal studies are needed to confirm the association between HBP and HMOD (kidney, in particular).
3. The respective role of morning vs. evening HBP with regards to outcomes is still unclear.
4. There is no convincing evidence on superiority of either HBP or ABP in predicting outcome, because of the lack of head-to-head comparisons.
5. There is still uncertainty on how to validate devices in large arms, sized greater than 42 cm, as there are issues with the cuff size and shape of the reference (auscultatory) BP measurement as well.
6. Lack of availability of properly sized cuffs, which can end up in an over estimation of BP whilst using a standard size cuff on large arms.
7. Established validation standards have not yet been developed to assess cuffless devices (ISO standard).
8. The use of HBP telemonitoring deserves further research to clearly demonstrate its clinical efficacy and economic benefits.
9. How to integrate self monitoring with clinical records, and most of all, how to choose patients for self-titration.
10. HBPV – what are the threshold values for risk stratification? Are there any independent prognostic benefits from reducing BPV? What treatments are effective in treating increased BPV?
11. Can HBPM reimbursement improve BP control in the populations?
12. More home BP devices should be validated in special populations (children, pregnant women, CKD, etc.).
13. Evidence-supported thresholds for hypertension in children and during pregnancy based on home BP measurements is needed.
14. Outcome data on the use of self-monitoring of BP for guiding management in children and in pregnancy is needed.
15. Nocturnal HBPM: clinical and prognostic value of HBPM devices for the detection of nondippers and of sleep apnea patients, and usefulness/reliability of wrist-worn nocturnal BP monitors is to be established.
16. Randomized controlled intervention trials exploring whether hypertension management based on HBPM leads to a better outcome than a management based on OBP are needed. The ongoing MASTER trial [275] is not only exploring this important issue with regard to ABPM but also includes data on HBPM and might provide some evidence in this regard.

## 16. CONCLUSION

Compared with the last 2008–2010 Position Papers [4], the number of articles published in the field of HBPM in the last decade has been on considerable rise and convincing evidence has now further clarified several aspects about the use of HBPM in clinical practice and research. Some of these new indications are summarized in Boxes 31 and 32. Additional evidence is still needed from population studies and randomized trials on hypertension management, to clarify whether hypertension management based on out-of-office BP, in particular on HBPM, leads to a better outcome than hypertension management guided by OBP.

Box 31 **New indications on methodology of HBPM**

- The use of wide-range cuffs with oscillometric devices is useful for HBPM, (but cuff choice should be based on the instruction by the manufacturer)
- A new Universal Standard for validation of BP monitors has been published in 2018 [90]
- The main international website providing a reference list of validated devices is now www.stridebp.org
- Cuffless devices may offer information on BP in a less intrusive manner, but to date, none of them has been properly validated
- Main characteristics of a preferred HBPM device include: upper arm cuff; successful validation within the last 10 years and storage/connectivity for objective reporting of readings
- Use of such devices is recommended both for clinical and research purposes

Box 32 **New indications on clinical application of HBPM**

- Re-definition of diagnostic thresholds for hypertension: HBP at least 135/85 mmHg corresponds to at least 140/90 mmHg clinic BP in ESC-ESH guidelines), whereas HBP at least 130/80 mmHg may correspond to at least 130/80 mmHg clinic BP threshold for grade I hypertension in ACC/AHA guidelines [2]
- Therapeutic targets: systolic HBP between 125–130 mmHg and diastolic HBP less than 80 mmHg are now considered reasonable goals. Such targets do not apply in the very elderly where higher systolic HBP values might be considered for SBP
- Recent technologies have now made nocturnal HBPM feasible. Studies are needed to explore whereas nocturnal HBPM can improve the prognostic stratification of patients with hypertension

## ACKNOWLEDGEMENTS

All authors thank OMRON Healthcare and SERVIER for the generous and unrestricted support to the organization of the ESH Satellite Symposium held in Milan on June 2019, from which the present work was generated.

### Conflicts of interest

R.A. has received honoraria from Akebia, AstraZeneca, Bayer, Boehringer Ingelheim, Chinook, Diamedica, Merck, Reata, Relypsa and Sanofi, and has received royalties from UpToDate. R.A. is supported by National Heart Lung and Blood Institute grant R01 HL126903 and U.S. Veterans Administration grant I01CX001753. K.A. has received Research support from Omron Healthcare.

P.G. has received Research grants from Recor Medical. K.K. has received Research grants from Omron Healthcare Fukuda Denshi, and A&D, Otsuka Medical. Y.L. has received research grants from A&D, Bayer, Omron and lecture fees from A&D, Daiichi Sankyo, Omron and Takeda. J.M. is part of the Scientific Advisory Board for BMS/Pfizer. R.J.M., grant income from NIHR; telemonitoring development and evaluation with Omron (all fees to his institution); speaker expenses ESH 2019 and ASN 2019. Honoraria paid to his institution/College. A.S.M. has received research funding from Novartis & honorarium from Merck, Servier, Hillrom. T.O. has received research grant from Omron Healthcare Co.Ltd. S.O. is a scientific consultant of Biotechmed Ltd. provider of telemedicine services. G.P. has received honoraria for lectures by OMRON Healthcare, SERVIER, Sanofi, FIDIA.

P.P. has received honoraria for validation Studies from MIcrolife, AND, and Hingmed. G.S. has conducted validation studies for various manufacturers of blood pressure measurement technologies and advised manufacturers on device and software development. J.-G.W. has received grants from Omron. M.A.W. is a consultant for Medtronic; ReCor; Ablative Solutions; Urovant; Regeneron; Bayer.

W.B.W. is a cardiovascular safety consultant (DSMB, CV endpoints committees) for Astra-Zeneca, Bristol-Myers Squibb, Takeda-Millenium; and editor for UptoDate (Wolters Kluwer).

## Supplementary Material

Supplemental Digital Content
