# Supplementary material for: Home blood pressure monitoring: methodology, clinical relevance and practical application: a 2021 position paper by the Working Group on Blood Pressure Monitoring and Cardiovascular Variability of the European Society of Hypertension
Source: J Hypertens. 2021 Jul 26;39(9):1742–67. doi: 10.1097/HJH.0000000000002922 (PMC9904446; doi:10.1097/HJH.0000000000002922)
Supplement: Supplemental Digital Content [file jhype-39-1742-s001.docx]

**HOME BLOOD PRESSURE MONITORING: METHODOLOGY, CLINICAL RELEVANCE, AND PRACTICAL APPLICATION.**

**A 2021 Position Paper by the European Society of Hypertension.**

**Working Group on Blood Pressure Monitoring and Cardiovascular Variability.**

**ONLINE ONLY SUPPLEMENTAL MATERIAL**

**Parati G,^1,2^ Stergiou G,^3^ Bilo G,^1,2^ Kollias A,^3^ Pengo M,^1^ Ochoa JE,^1^ Agarwal R,^4^ Asayama K,^5,6,7^ Asmar R,^8^ Burnier M,^9^ De La Sierra A,^10^ Giannattasio C,^2,11^ Gosse P,^12^ Head G,^13^ Hoshide S,^14^ Imai Y,^7^ Kario K,^14^ Li Y,^15^ Manios E,^16^ Mant J,^17^ McManus RJ,^18^ Mengden T,^19^ Mihailidou A,^20^ Muntner P,^21^ Myers M,^22^ Niiranen T,^23,24^ Ntineri A,^3^ O’Brien E,^25^  Octavio A,^26^ Ohkubo T,^5,7^ Omboni S,^27,28^ Padfield P,^29^ Palatini P,^30^ Pellegrini D,^31^ Postel-Vinay N,^32^ Ramirez AJ,^33^ Sharman JE,^34^ Shennan A,^35^ Silva E,^36^ Topouchian J,^37^ Torlasco C,^1^ Wang J,^15^ Weber M,^38^ Whelton PK,^39^ White W,^40^ Mancia G.^41^ on Behalf of the Working Group on Blood Pressure Monitoring and Cardiovascular Variability of the European Society of Hypertension.**

1-Istituto Auxologico Italiano, IRCCS, Department of Cardiovascular Neural and Metabolic Sciences, Milan, Italy

2-Department of Medicine and Surgery, University of Milano-Bicocca, Milan, Italy

3-Hypertension Center STRIDE-7, National and Kapodistrian University of Athens, School of Medicine, Third Department of Medicine, Sotiria Hospital, Athens, Greece

4-Division of Nephrology Department of Medicine, Indiana University School of Medicine and Richard L. Roudebush Veterans Administration Medical Center, Indianapolis, IN, USA.

5-Department of Hygiene and Public Health, Teikyo University School of Medicine, Tokyo, Japan.
6-Research Unit Hypertension and Cardiovascular Epidemiology, KU Leuven Department of Cardiovascular Sciences, University of Leuven, Leuven, Belgium.
7-Tohoku Institute for the Management of Blood Pressure, Sendai, Japan

8-Foundation-Medical Research Institutes, Geneva, Switzerland

9- Service of Nephrology and Hypertension, University Hospital, Lausanne, Switzerland

10-Hypertension Unit, Department of Internal Medicine, Hospital Mútua Terrassa, University of Barcelona, Barcelona, Spain.

11-Cardiology IV, ‘‘A.De Gasperis” Department, ASTT GOM Niguarda Ca’ Granda

12- Cardiology/Hypertension Unit Saint André Hospital. University hospital of Borfeaux, France

13-Baker Heart and Diabetes Institute, Melbourne Victoria Australia

14-Division of Cardiovascular Medicine, Department of Medicine, Jichi Medical University School of Medicine, Tochigi, Japan

15-Shanghai Institute of Hypertension, Ruijin Hospital, Shanghai Jiaotong University School of Medicine, Shanghai, China

16-Department of Clinical Therapeutics, National and Kapodistrian University of Athens, School of Medicine, Alexandra Hospital Athens, Greece

17-Primary Care Unit, Department of Public Health & Primary Care, University of Cambridge, Cambridge, UK

18-Nuffield Department of Primary Care Health Sciences, University of Oxford, Oxford OX2 6GG.

19-Kerckhoff Clinic, Rehabilitation, ESH Excellence Centre, Bad Nauheim, Germany

20-Department of Cardiology and Kolling Institute, Royal North Shore Hospital, St Leonards Faculty of Medicine and Health Sciences, Macquarie University, Sydney, Australia 2065.

21-Hypertension Research Center, Department of Epidemiology, School of Public Health, University of Alabama at Birmingham, Birmingham, AL, USA.

22- Schulich Heart Program, Sunnybrook Health Sciences Centre and Department of Medicine, University of Toronto

23- Department of Medicine, Turku University Hospital and University of Turku, Finland

24- Finnish Institute for Health and Welfare, Helsinki, Finland

25-The Conway Institute, University College Dublin, Dublin 4. Ireland

26-Experimental Cardiology, Department of Tropical Medicine Institute, Universidad Central de Venezuela

27-Clinical Research Unit, Italian Institute of Telemedicine, Varese, Italy.

28-Department of Cardiology, Sechenov First Moscow State Medical University, Moscow, Russian Federation

29-Department of Medical Sciences, University of Edinburgh, Edinburgh, United Kingdom

30-Department of Medicine. University of Padova, Padua, Italy

31-Cardiovascular Department ASST Papa Giovanni XXIII, Bergamo, Italy

32-Hypertension Unit. European Georges Pompidou Hospital. Paris, France.

33-Arterial Hypertension and Metabolic Unit, University Hospital, Fundacion Favaloro.

34-Menzies Institute for Medical Research, College of Health and Medicine, University of Tasmania, Hobart, Australia

35-Department of Women and Children’s Health, School of Life Course Sciences, FoLSM, Kings College London.

36-Research Institute of Cardiovascular Diseases of the University of Zulia, Venezuelan Foundation of Arterial Hypertension. Maracaibo, Venezuela

37-Diagnosis and Therapeutic Center, Paris-Descartes University, AP-HP, Hotel Dieu, Paris, France

38-Division of Cardiovascular Medicine, Downstate College of Medicine, State University of New York, Brooklyn, New York, USA

39-Department of Epidemiology, Tulane University, School of Public Health and Tropical Medicine

40-Cardiology Center, University of Connecticut School of Medicine, Farmington, CT, USA

41-University Milano-Bicocca, Milano, Italy

The present document has been developed by the *ESH Working Group on Blood Pressure Monitoring and CardioVascular Variability,* with input from an international team of experts, and has been endorsed by the ESH.

As acknowledged in several hypertension clinical practice guidelines, including the 2017 American College of Cardiology (ACC)/American Heart Association (AHA) (1) and the 2018 European Society of Cardiology (ESC)/European Society of Hypertension (ESH) guidelines, (2), management of hypertension is sub-optimal when based only on office or clinic blood pressure (BP) measurements. This has stimulated the introduction of out-of-office blood pressure monitoring methods, including ambulatory (ABPM) and home BP monitoring (HBPM), which have been increasingly used in clinical practice over the last decades, with a favourable impact on hypertension diagnosis and BP control.

Adoption of HBPM, in particular, has had an exponential growth, favoured by technological progress which has led to the availability of small, accurate, user-friendly and relatively inexpensive BP monitoring devices. Such an important growth has highlighted the need to provide instructions to both patients and doctors for an optimal application of self-BP monitoring at home in daily practice and in clinical research.

The current update has taken into account new evidence in this field, including a recent statement by the AHA (3), as well as technological developments which have occurred over the past decade.

Hypertension guidelines have to deal with a large number of complex issues and their scope is to provide recommendations on all or most diagnostic and treatment aspects of a chronic BP elevation. This broad scope limits the space for a detailed discussion and specific recommendations on practical aspects of BP measurement, despite their crucial role for hypertension diagnosis, estimation of cardiovascular risk and evaluation of the effects of treatment. This has been the case for HBPM as well, which is recommended in recent hypertension guidelines but without detailed instructions on its practical application. These instructions are provided in the present manuscript, together with an update on the emerging technologies in this field in order to provide healthcare professionals with guidance that details the appropriate use of contemporary HBPM in clinical practice and research (2), (1), (4), (5), (6), (7), (8), (9), (3).

**S.6.1-Reproducibility of HBPM**

Chatellier *et al* examined the SD of the differences between two HBPM sessions derived from increasing numbers of triplicate morning and evening BP measurements over two 10-day intervals in 79 untreated individuals (10) and showed that 80% of the maximal reproducibility (reduction in SD of differences) was obtained by averaging 15 measurements over the initial 5 days. (10) In another study from the same group, 1,710 hypertensive patients measured their BP three times in the morning and evening over 4 days and discarded the first day. (11) The study showed that by increasing the number of measurements from 1 to 18, the SD of SBP/DBP was reduced by 17%/23%. (11) Regarding the long-term reproducibility over a 1-year period, a study in 136 untreated subjects showed HBPM (3 or more days) to be superior to office BP measurement. (12)

The reproducibility of home pulse pressure was investigated using the SD of differences between measurements in 393 hypertensive subjects who had repeated office, home and ambulatory BP measurements. (13) The SD of differences between repeated measurements was 5.2 mmHg for home pulse pressure (2 days) compared to about 10 mmHg for single-visit office and 4 mmHg for 24-hour ambulatory pulse pressure. (13)

The reproducibility of morning versus evening home BP measurements has also been investigated. A study showed no differences in the clinical evaluation made using any set of morning home BP measurements over a short period (7 days), yet with superior reproducibility than evening measurements. (14) Another study showed morning home BP to have superior reproducibility and closer association with vascular indices than ambulatory BP. (15) The reproducibilty of Home BP tended to be superior (and not similar) to ABPM as indicated by test-re-test correlations in the study by Stergiou et al (16) (n=133): Home BP =0,91/0,86, Office BP=0,77/0,76, ABPM =0,80/0,84. SD of mean differences : Office BP =11.0/6.6, day-time-ABP =10.0/6.6, Home BP =6.9/4.7. In the study by Uen et al in 2009 (n=97) these results were confirmed, with SD and 95% CI for systolic Home BP = 3,81 (3,34-4,44), for ABP =7,83 (6,9-9,17) and for Office BP =8,1 (7,1-9,43). (17)

The 2003 and the 2012 Japanese Society of Hypertension Guidelines for Self-Monitoring of Blood Pressure at Home, (18), (19) were the first guidelines for home BP monitoring emphasizing the first priority of home BP in the diagnosis and management of hypertension. A recent [Expert panel consensus recommendations for home blood pressure monitoring in Asia has been issued by the Hope Asia Network.](https://pubmed.ncbi.nlm.nih.gov/29386668/)  (20)

The reproducibility of hypertension diagnosis is also better with HBPM than with OBP measurements. In a study on untreated individuals the agreement rate for the diagnosis of masked hypertension over a 1-week period appeared to be higher for HBPM (82%) than 24-hour (68%) or awake ambulatory BP (71%). (21) In another study, OBP measurements over 7 visits and HBPM over 1 week exhibited a similar low persistence of white-coat and masked hypertension diagnoses over 1 year. (22), (23) In a Japanese study in 503 untreated subjects, the reproducibility of masked hypertension diagnosis over a 6-month period was superior when evaluated by morning than evening home BP (24).

On such a background the 2014 Guidelines of the Japanese Society of Hypertension recommended for the first time that "when there is a discrepancy of diagnosis between OBP and HBP, a HBP-based diagnosis should have priority" (25) . This recommendation is taking over in the latest 2019 version of these guidelines (26).

**S6.2- Diagnostic ability**

**S6.2.1-Diagnostic accuracy and Identification of MH and WCH**

Current guidelines for hypertension management around the world have attributed an important role to ABPM and HBPM for an accurate diagnosis and management of hypertension (2), (1), (4), (5), (6).

The 2019 UK NICE guidelines recommend ABPM as the most cost-effective strategy for confirming the diagnosis of hypertension, with HBPM used when ABPM is unsuitable or intolerable (4), (5). The 2017 ACC/AHA guidelines give a primary role to HBPM, particularly in treated adults with hypertension because of its greater practicability, much wider availability in primary care settings, and its better acceptance by users for long-term application (1). The 2018 ESC/ESH guidelines recommend out-of-office BP monitoring, or when this is not feasible, repeated OBP measurements at several visits, to confirm the diagnosis of hypertension~~.~~ The guidelines present a list of advantages, limitations and clinical indications of either HBPM and ABPM, and consider them complementary rather than alternative methods (2), (7), (8).

Systematic reviews of studies assessing the diagnostic accuracy of HBPM have typically taken ABPM as reference method and have reported moderate diagnostic agreement between ABPM and HBPM (kappa 0.40-0.60), with higher specificity and negative predictive value (70-90%) and lower sensitivity and positive predictive value (60-80%) of HBPM in most studies (27), (28).

Meta-analysis of 3 studies comparing home with ambulatory monitoring showed sensitivity 86% and specificity 62% of HBPM for hypertension diagnosis. (29) The diagnostic performance of HBPM appeared to be similar for different hypertension phenotypes and in untreated and treated individuals assessed in these studies. (6), (27), (28) Moreover, there was a clear superiority of HBPM compared to office measurements in diagnosing uncontrolled hypertension and assessing the effects antihypertensive treatment. (27)

The China Ambulatory and Home BP Registry (N=1,774) showed HBPM to have high specificity, but low sensitivity in diagnosing white-coat and masked hypertension, implying that HBPM and ABPM are not interchangeable but rather have a complementary role. (30), (31) In the PAMELA (Pressione Arteriose Monitorate E Loro Associazioni) outcome study in 2,051 individuals in Italy the diagnostic disagreement between HBPM and ABPM was 22%. (32) In a cross-sectional analysis of a European dataset there was a diagnostic disagreement between HBPM and ABPM in 20% of 1,971 individuals, which was most likely due to methodological and patient-related factors. (33) However, when cases with clinically irrelevant HBP-ABP difference or with diagnostic uncertainty (BP difference ≤5 mmHg from threshold) were excluded, the ‘certain’ disagreement between the two methods was reduced to 8%. (33)

In a European dataset with 1,971 participants, among 445 individuals with masked hypertension 48% had masked hypertension with both methods, 30% had isolated ambulatory masked hypertension and 22% isolated home masked hypertension. (34) In another study in the USA in 333 community-dwelling untreated adults with clinic BP <140/90 mm Hg, 30% had masked hypertension with both methods, 61% isolated ambulatory and 9% isolated home masked hypertension. (35) In the latter study after multivariable adjustment and compared with participants without masked hypertension on ABPM and HBPM, those with dual or isolated ambulatory masked hypertension had higher left ventricular mass index, whereas those with isolated home masked hypertension did not have increased left ventricular mass index.(35) Outcome studies have shown that individuals with masked or white-coat hypertension identified only by HBPM or only by ABPM have intermediate cardiovascular risk between those identified by both methods as normotensives and hypertensives respectively, which supports the complementary role of the two methods in defining prognosis. (36), (37) Mancia et al showed increasing outcome prediction ability by increasing the number of BP measurement methods from 1 to 3. (32)

Studies assessing *morning BP* showed similar results using either HBPM or ABPM. (38), (15)

A study in 1,049 untreated individuals showed morning home BP to be more reproducible and more closely associated with vascular indices than morning ambulatory BP. (15)

In a study in 333 Veterans with chronic kidney disease (CKD) the prevalence of MUCH using three definitions of out-of-office hypertension on the basis of either 24-hour ABPM ((i) daytime hypertension ≥135/85 mmHg, (ii) either nighttime hypertension ≥120/70 mmHg or daytime hypertension, and (iii) 24-hour hypertension ≥130/80 mmHg) or HBP monitoring (hypertension ≥135/85 mmHg), was 26.7% with daytime ABP, 32.8% with 24-hour ABP, 56.1% with daytime or nighttime ABP and 50.8% with HBP. (39) Prevalence of MUCH increased with increasing clinic systolic BP: 2% in 90-110 mmHg group, 17% in BP 110-119 mmHg group, 34% in 120-129 mmHg group, and 66% in 130-139 mmHg group. Clinic BP was a good determinant of MUCH (ROC AUC 0.815 (95% CI 0.758--0.872)). In diagnosing MUCH, home BP was no different from ABP. In veterans with chronic kidney disease (CKD) not on dialysis, the agreement in the diagnosis of MUCH by ABP tested 4 weeks apart was between 75% and 78% (kappa coefficient for agreement (0.441 to 0.509). In contrast, HBP showed an agreement of 63% and kappa coefficient of 0.249 (39) A study conducted in a large registry on elderly non-valvular AF patients, a [High prevalence of masked uncontrolled morning hypertension](https://pubmed.ncbi.nlm.nih.gov/33190415/) was reported (40)

### **S6.3-Prognostic value of HBPM**

### **S6.3.1-Hypertension mediated organ damage (HMOD)**

***Cardiac damage***

In some studies comparing the correlation of HBPM and ABPM with cardiac damage, home BP showed closer association with LVMI than ambulatory BP. (41), (42) Also, electrocardiographically detected left ventricular hypertrophy (LVH) was reported to be associated with HBP and this association was stronger than with office BP. (43), (44), (45) Finally, in Finn-Home study the prevalence LVH detected by ECG was higher in subjects with masked hypertension detected with HBP than in normotensive participants. (46) Associations of HBP with other indices of cardiac HMOD were also reported including left atrial diameter (47) and volume. (42)

Few longitudinal studies on changes in LVM in relation with HBP are available. Tsunoda et al. reported LVMI increase in subjects with poor or worsened home systolic BP but no association with office BP changes. (43) Similarly, in the Study on Ambulatory Monitoring of Blood Pressure and Lisinopril Evaluation (SAMPLE) treatment-induced reductions in home BP correlated slightly better with treatment-induced changes in LVMI than reductions in office BP (but not as well as changes in ambulatory BP). (48) Interestingly, in the PAMELA study normotensive subjects with LVH at baseline had higher incidence of hypertension on HBP (and ABP) over follow-up, which casts some doubt on the cause-effect relationship between increased out-of-office BP and LVH. (49) In the latter two studies, however, HBP protocol involved few measurements and therefore their results should be interpreted with caution. In Finn-Home study, changes in HBP were correlated with changes in ECG parameters used to identify LVH and this correlation was closer than for office BP changes. (50)

***Vascular Damage***

In another study HBP was independently associated with cIMT also after adjusting for OBP level (51). Association of masked hypertension detected by HBP with the risk of carotid atherosclerosis was also reported in several studies. In the Hisayama study increased cIMT was associated with masked hypertension and similarly with white coat hypertension (52), while masked hypertension but not white coat hypertension was related to increased cIMT or the presence of plaques (53). Similar results were reported in the Finn-Home study. (46)

Meta-analysis of three studies did not find differences between HBP and OBP in terms of their association with carotid-femoral or aortic-popliteal pulse wave velocity (PWV) (54). In the Finn-Home study higher aortic PWV was observed in masked hypertension defined by HBP versus normotensives but not versus white coat hypertensive participants (46). Finally, in a study of Matsui et al. (55) aortic PWV was higher in masked and masked uncontrolled hypertensives, while white coat hypertension was associated with increased aortic stiffness only in untreated participants.

***Kidney Damage***

Regarding the association of HBP with glomerular filtration rate (GFR), the available data are very heterogeneous and comparisons of HBP and OBP are not available. In the Indianapolis study, GFR was no longer a determinant of BP once urine protein excretion was accounted for (56).

Associations of masked hypertension detected with HBP with kidney damage have been reported. In the previously mentioned Ohasama study incident albuminuria and chronic kidney disease (CKD) were associated with masked but not with white coat hypertension. Conversely, in the Hisayama study white coat hypertension was equally associated with UACR as masked hypertension and no differences between BP phenotypes were found for GFR (57). Masked uncontrolled hypertension is more strongly related to albuminuria compared with cardiovascular damage as assessed by left ventricular mass and PWV. A graded and an independent relationship of BP classification status with albuminuria was seen (58).

**S6.3.2-Adverse health outcomes**

**Cardiovascular events**

It has been suggested that morning HBP might be prognostically superior to daily or evening values (59), (60), but most of this evidence is derived from Japanese studies. Because of substantial differences between BP phenotype in Japan and in other Countries/Regions, it is not clear if these findings can be extrapolated to other populations.

An added prognostic value of HBPM on top of that provided by OBPM is evident in studies on masked/masked uncontrolled hypertension (MH/MUCH) and white coat hypertension (WCH) detected with HBP. In the IDHOCO database MH was associated with adverse outcomes (fatal and nonfatal events) both in untreated and treated participants (HR 1.55; 95% CI [1.12-2.14] and 1.76; 95% CI [1.23-2.53], respectively), while WCH conveyed increase of risk in untreated subjects but not in those on antihypertensive treatment (1.42; 95% CI [1.06-1.91] and 1.16; 95% CI [0.79-1.72] (61) respectively. Similar results have been reported in recent meta-analyses (62) (63, 64). In the Japanese general practice population, masked hypertension defined by HBPM appeared to be associated with an increased risk for stroke events (65).

There is limited evidence regarding the HBP association with specific types of events but the available data suggest this association is strongest for stroke, while in case of coronary events it may be restricted to untreated individuals (66), (60), (65).

There are limited data also on longitudinal outcomes relating HBP to kidney failure outcomes. In a study of US Veterans, 1 standard deviation increase in home systolic BP increased the risk of end-stage kidney disease (ESKD) by 1.74 (95% CI 1.04–2.93) when adjusted for standardized clinic systolic BP, proteinuria, estimated glomerular filtration rate, and other risk factors (67). This study found that among patients with CKD, BPs obtained at home are a stronger predictor of ESKD or death compared to BPs obtained in the clinic.

Evidence has been provided that nocturnal hypertension defined by home BP monitoring is a better predictor of cardiovascular events than that by ABPM. (68)

**S6.4.1-Improving adherence to treatment**

In a recent study, use of HBPM by patients was shown to be higher among those given a physician recommendation, emphasizing the role for physicians in counseling and partnering with patients on HBPM use for BP management (69).

In any case, HBPM is recommended as a means to increase treatment adherence and persistence and to stimulate other lifestyle changes because of a deeper engagement with the patient leading to a better control of this risk factor. Its use might help in case of the so called “white coat compliance”, a condition where a given patient starts taking the prescribed pills only when the time of a scheduled clinic visit is approaching, and stop doing so right after it (70).

**S7.1.1-Types of cuff-based devices for HBPM**

HBPM devices are either manual, semi-automated, or automated. Semiautomated devices are characterized by manual cuff inflation and automatic deflation; automated devices are characterized by automatic cuff inflation and deflation. The accuracy of oscillometric devices usually remains stable over time and, therefore, they do not require frequent calibration. Among the validated HBPM devices, there are now several options to consider.

- - The simplest devices require the user to press a button to initiate a reading, which is then displayed after the reading is taken.
  - Some devices can be programmed to take 2 or 3 readings with the option of specifying the interval between readings (eg, 1 or 2 minutes).
  - Devices whose readings can be automatically stored and averaged, in case of a single user or with a function to store separately users’ readings (with the dates/times they were taken), displayed on the device screen, printed, or transmitted to cloud or the healthcare provider, should be preferred (www.stridebp.org/bp-monitors).
  - Some devices can specifically detect atrial fibrillation (AF) with high diagnostic accuracy, although there are no consistently accurate devices for AF and other arrhythmias.
  - A few devices allow automated prescheduled nighttime BP monitoring (nocturnal mode)
  - Monitors that are more than 4 years old are less likely to remain accurate. (71)

**Devices for nocturnal HBPM**

Studies have been specifically conducted in order to compare a supine algorithm-equipped wrist nocturnal home blood pressure monitoring device with an upper arm device. (72)

**S7.2-Cuffless devices**

In recent years, several prototypes of cuffless devices have been proposed, based on a variety of different methods for BP estimate. (73), (74), (75), (76), (77), (78), (79), (80), (81), (82), (83), (84), (85) At present, however, convincing evidence is still missing to support their use in clinical practice.

Different methods for cuffless BP measurement have been developed, including tonometry, pulse wave velocity, pulse transit time, pulse wave analysis and **plethysmography.** The latter approach measures volume changes that are transformed into changes of pressure with calculation of systolic and diastolic BP and pulse rate values according to specific algorithms. Accuracy of most plethysmography-based cuffless devices for BP measurements which might be used for HBPM remains controversial. In fact, to our knowledge, none of these very popular devices (watches, bracelets, smartphone Apps) satisfy regulatory requirements or has been validated according to currently established protocols. Moreover, some of these devices require initial calibration with a reference method, and there are pending issues regarding long-term persistence of their calibration, especially under varying behavioural conditions.

Therefore, despite their large distribution mainly as multiple parameters monitoring bracelets or watches, the use of these devices is not presently recommended for HBPM as their accuracy and reliability remains highly questionable. Principles of **tonometry** for measuring radial BP and performing pulse wave analysis using a transfer function has been reported and described in detail previously elsewhere. (86), (87), (88), (89) Briefly, tonometry means “measurement of pressure” whereas applanation means “to flatten” the arterial wall. Applanation tonometry is performed by placing one or several tonometers (strain gauge pressure sensor) over the radial artery and by applying soft pressure to obtain an assumed flattened arterial wall. This method was designed for clinical use by researchers particularly to measure the radial BP and calculate aortic (central) BP by performing the pulse wave analysis and using algorithms such as a transfer function. (86), (87), (88), (89) Considering the importance of aortic BP, manufacturers have tried to extrapolate the use of this technique for HBPM, but this approach is still under development and at this time remains reserved for research.

Several other techniques to measure BP have been proposed for HBPM such as deriving a BP estimate from **pulse transit time assessment**. This technique is based on the assessment of pulse wave velocity and on use of its reciprocal variable, the pulse transit time, to calculate beat-by-beat BP values through a dedicated algorithm, following an initial calibration through a conventional arm cuff inflation-based measurement. Preliminary validation studies of this approach suggest short term accuracy, up to 30 min after calibration (90), while long term validation data are missing or yet unsatisfactory, in particular for BP monitoring during night sleep. Lastly, it is worth mentioning **smartphone apps which use the light absorption changes from a finger to estimate changes in blood volume and to calculate finger BP** values by considering the relationships between changes of blood volume and the corresponding changes in BP. None of these techniques can be currently recommended as a reliable method for performing HBPM.

**S7.3.2-New approaches to HBPM telemonitoring. Mobile Health: current evidence, future perspectives**

Despite the promising results and future perspectives of mHealth related interventions there are still some issues in digital Health that should be addressed.

-Due to the fast-paced growth of this field and the **absence of clear and strong regulations**, in 2015 the US Food & Drug Administration (FDA) released guidance recommendations for the developers and distributors of health-related apps, to establish specific requirements

(http://www.fda.gov/downloads/medicaldevices/deviceregulationandguidance/guidancedocuments/ ucm263366.pdf) but worldwide regulations are still insufficient.

-**Accuracy of the scientific content** of the services is another crucial topic. All devices and applications must have a proper scientific validation. Smartphone applications frequently provide additional features, like educational sections for patients, or decisional tools for physicians, which also need validation.

-Even more advanced eHealth systems for HBPT still depend on traditional **BP measurement** with a cuff, as cuffless devices are not yet accurate enough for a real daily employment in clinical practice.

Therefore, reliable performance of the m-health based strategies requires attention to a number of methodological issues, the most important still being the use of validated BP measuring devices. There is a strong need for adequately powered randomized controlled trials, to address the efficacy, feasibility and cost-effectiveness of these new strategies, especially in the field of mHealth, with a close collaboration between industry and academic institutes, aimed at developing better devices and useful tools for physicians and patients.

Evidence from a recent clinical trial in individuals with uncontrolled hypertension, showed that those randomized to a smartphone coaching app (i.e. to promote home monitoring and behavioral changes associated with hypertension self-management) plus home monitor had similar systolic BP compared with those who received a BP tracking app plus home monitor (91).

Although available studies suggest a beneficial effect on BP control by these novel HBPT technologies, the high heterogeneity of proposed interventions and the lack of standardization of available trials are a strong limitation to the formulation of recommendations based on solid evidence.

**S8.1-Optimal monitoring schedule**

By averaging more HBP readings, the average HBP value and its variability are both reduced, with most of this decline occurring over the initial 6–15 measurements over the first 3 monitoring days (92), (28), (10), (11) (93), (16), (94), (95), (96). The Finn-Home study showed: that (i) there is no significant effect of discarding the first day of a 7-day schedule, (ii) morning and evening BP have similar predictive ability, and (iii) duplicate compared to single HBP measurements on each measurement occasion have a small benefit in predictive value (97). A recent systematic review showed that increasing the number of HBPM days improves its prognostic ability (72%-91% of the theoretical maximum predictive value reached by 3 days and 86%-96% by 7 days) (98). However, there was no convincing evidence that the timing or number of readings per day, or discarding the first day influenced these findings (98).

A study in 56 patients showed that with 1-min intervals between HBP readings the agreement with daytime ABPM was closer than with 10-s interval (99)

**S8.3 Therapeutic targets and treatment titration**

**Therapeutic targets**

The 2008 ESH guidelines on HBP, while acknowledging the lack of direct evidence, concluded that the target HBP for therapy should logically be below the threshold used to diagnose hypertension, that is less than 135/85 mmHg for systolic/diastolic HBP. At that time a few studies focusing on HBP targets were available, namely THOP and HOMERUS but they did not address the HBP targets effect on outcomes, applied the same BP targets for OBP and HBP and had several further methodological problems (100), (101), (102).

A randomized study in hypertensive patients regarding the strategy for treatment initiation and titration using either HBP alone, or OBP and ABP, found no difference in terms of organ damage regression after 1 year (103).

Although no evidence from interventional studies is available to identify HBP targets in the elderly, the results of the observational IDHOCO study indicated that in octogenarians taking antihypertensive medication the lowest risk was associated with systolic HBP of 148.6 mmHg, whereas diastolic BP values ≥82 mmHg minimized risk (diastolic BP ≤65.1 mm Hg entailed increased cardiovascular risk) (104).

**Treatment Titration**

Treatment titration and long term follow up is the most important domain of home BP.

The TASMINH4 study aimed to assess both the longer term (12 month) effect of titration using HBPM and the influence of telemonitoring over and above HBPM with simple paper-based feedback on hypertension control. Overall, 1182 hypertensive patients aged over 35 with uncontrolled BP were randomised to one of three groups: either hypertension management based on clinic readings or HBPM (using paper charts to record BP with monthly posting of results to the GP or HBPM with a simple text-based telemonitoring system). After 12 months, both HBPM groups had significantly lower systolic BP than those titrated based on clinic readings and the telemonitoring group also had lower BP at 6 months suggesting quicker titration with telemonitoring. (105) (106). TASMINH4 thus showed (unlike THOP and HOMERUS) that BP control was better with self-monitoring (105).

Taken together, these data suggest that physicians using HBPM to titrate antihypertensive medication can achieve better hypertension control than when only OBP is used. The key factor for success seems to be a target of <135/85 mmHg for home readings and using self-BP monitoring on a regular basis in a structured way. If a telemonitoring system is used it should be simple, with feedback to both professionals and patients. Patients should be given a choice regarding the use of telemonitoring, however.

Another approach tested even before the first studies on professional titration using self-monitored BP was the one based on Patient Self-Monitoring with Self-Titration.

After the first studies showing promising results (107), 10 years later the TASMINH2 trial confirmed that patients randomised to HBPM with self-titration and followed-up for a year exhibited 5.4/2.7 mmHg lower BP compared to controls (108).

Indication in this regard provided by Different Guidelines are shown in table S2. A flow chart with indications on use of HBPM and ABPM in Hypertension Management and Treatment Titration is provided in Figure S1

**S8.4-HOME vs. ABPM**

As already mentioned, in spite of the routine use of OBP measurement as a cornerstone in hypertension management, OBP represents only a screening technique, with out-of-office measurements being required in most cases before diagnostic or therapeutic decisions are made (109), (110), (111), (112), (113), (114), (115), (1). The rationale for this recommendation is based on the fact that OBP monitoring is subject to major limitations that make it unrepresentative of the true BP. This is because 1) not many readings are obtained and 2) the doctor’s office is an artificial environment that lacks ecological validity. Moreover, OBP measurements alone cannot identify clinically relevant conditions such as white-coat hypertension (misleadingly high office measurements vs normal out-of-OBP) and masked hypertension (misleadingly low office readings vs elevated out-of-OBP) (116), (117),(111), (118).

**HBPM vs ABPM: Clinical Relevance**

- *White Coat and masked hypertension:* These patterns can be diagnosed using either ABPM or HBPM, but the prevalence of these conditions can be different when estimated with one or the other method. In a direct comparison of these two methods, only half of the patients were defined as having masked hypertension on the basis of both ABPM and HBPM, whereas in the remaining ones, the diagnosis was made by one method but not by the other (119), (120). A relatively low (75%) agreement between ABPM and HBPM has been reported in the evaluation of poorly controlled hypertension, HBPM classifying a lower proportion of patients as having masked hypertension than ABPM (121)
- *Correlation with hypertension mediated organ damage (HMOD)*. Out of office BP measurements better correlate with HMOD than OBP. Direct comparisons of ABPM and HBPM did not identify significant differences in terms of correlation with left ventricular mass index or microalbuminuria, both being better than OBP (120), (121), (122), (123), (124)
- *Assessment of Treatment effects*. HBP monitoring alone is as reliable as combined clinic and ABP measurements in monitoring the effects of antihypertensive drug treatment on BP and preclinical target organ damage (103)
- *Mortality prediction.* The predictive value of selective and combined elevation in OBP, ABP and HBP for mortality was assessed in the PAMELA study: a selective elevation in HBP vs. ABP values or vice versa carried an increased risk. The overall ability to predict death, however, was not greater for HBP and ABP than for OBP. (125) ABP has similar or higher prognostic value than HBP, in particular when considering the prognostic value of nocturnal ABP (112), (126), (61)
- *Assessment of BP variability.* ABPM offers information on day-night and short term BP variations, while HBPM allows the assessment of day by day BP variability, which may contribute to outcome prediction. (127)

**S9.5-Patients with Chronic Kidney Disease (Box 24)**

In patients on haemodialysis, HBPM should be used to assess BP in the interdialytic period (128), (129), (130). Home BP relates to target organ damage (131), (132) and in one clinical trial to better interdialytic ABP control (133). Monitoring BP at home thus carries the potential to offer information useful to optimize management of volume and BP in patients on dialysis.

In such patients, it is recommended that HBP is measured twice daily, at bedtime and on waking, after the midweek dialysis for 4 days (134). This will allow sampling of a wide range of BP values which show satisfactory agreement with interdialytic 44-h ambulatory BP monitoring (135), (136).

In part due to volume accumulation, the mean change in systolic HBP in the interdialytic period is approximately 4 mmHg systolic/10 hours and this plateaus after the second interdialytic day (137). The diastolic BP does not increase as much; therefore the pulse pressure amplifies. Because of the marked BP fluctuations during dialysis, the peridialytic measurements are poorly reflective of the interdialytic BP behavior (138) and of the overall cardiovascular disease burden (138).

It is important to consider that patients on hemodialysis are never in steady state, and that BP increases progressively between 2 dialysis days. Patients treated with peritoneal dialysis should be monitored as other non-dialysis CKD patients, and this applies also to transplant patients.

**S10-Nocturnal Home Blood Pressure Monitoring**

In a substudy of the J-HOP comparing the prognostic value of nocturnal hypertension detected by HBPM versus ABPM, it was shown that the former was associated with increased risk of future cardiovascular events independent of office SBP, an association which was not found for nocturnal hypertension defined by ABPM (68).

Moreover, data from the J-HOP Nocturnal Blood Pressure Study, showed that masked nocturnal hypertension and sustained hypertension defined on the basis of nocturnal HBP, were associated with increased risk of CVD events (139).

**S12-Home blood pressure variability**

Regarding the optimal methodology of HBPV assessment two principal elements should be considered: 1) how HBP data are collected; 2) what estimates of HBPV should be considered.

Regarding the former, it should be acknowledged that most studies which reported on HBPV were designed to assess average HBP values. In Finn-Home Study HBPV was able to predict outcome when data from at least 3 days were used, with some minor improvement in predictive power when the monitoring was extended to 7 days. (140) Thus, the general standards for HBP monitoring methodology (including the choice of device and cuff, measurement conditions and schedule) should reasonably apply also to HBPV assessment.

As far as HBPV estimates are considered, they include measures of dispersion (Standard Deviation, SD; Coefficient of variation, CV; Variability Independent of the Mean, VIM), sequence-based measures (Average Real Variability, ARV; Interval Weighted SD, iwSD), and measures of instability (Range [Maximum-minimum BP], Peak size [Maximum BP], Trough size [Mean-minimum BP]). At the moment there is no clear evidence on the superiority of any of these indices when focusing on HBPV and its clinical value. Since HBPV is strongly related to average HBP values, which represent a potential confounder, measures of HBPV should be adjusted for average HBP in statistical analysis or normalized by calculating indices either independent from mean BP (e.g. “variation independent of mean”, VIM) or accounting for mean BP level (e.g. coefficient of variation, CV = SD*100/Mean BP).

At the moment there is no clear evidence that any treatment modality may be superior in reducing HBPV. In some small studies a reduction in HBPV was associated with the use of long-acting calcium antagonists (141), (142), (143) but these findings were not confirmed in the large HOMED-BP trial (144).

**S13.1 Barriers to clinical use of HBPM**

Diffusion of HBPM in developing countries may be severely limited by income levels of the population well below what occurs in more developed areas of the world. The high levels of poverty of a large part of the population in low-income countries does prevent mass dissemination of HBPM, since most of its inhabitants do not have the possibility of purchasing high quality and validated devices. Moreover, the low cultural level of a large part of the population does not guarantee that HBPM devices will be used in an appropriate manner as indicated by guidelines, and require a very important teaching effort by health care personnel. Even in low income countries patients who are able to pay for a healthcare service (the minority) may access ABPM and HBPM devices, their clinical implementation being managed in private centers, according to indications provided by Hypertension Guidelines. However, the majority of patients from low income countries are assisted in public hospitals or public health centers, free of charge or with a low contribution to health care costs. In these conditions the problem of devices availability has to be faced. In the case of ABPM, with availability of a moderate number of devices it might be possible to provide service to a significant percentage of hypertensive patients, with an effective organization of the clinic appointments, given that the ABPM device turnover is slightly longer than 24 hours. Thus implementation of 24h ABPM, in spite of the higher cost of each device, could be possible also in low resource settings, but only in presence fo an effective health cre organization on the contrary, although the equipment has a much higher cost, is in the hands of these centers, public or private, to install it to the patients for only one day, placing and withdrawing equipment, as well as analyzing the results, but with an important effort on the part of health personnel. Conversely, to achieve a generalized use of HBPM in low-resource settings, hypertension centers should have an enormous amount of devices available, to cover the clinical requirements of a large segment of the hypertensive population.

This being the current situation, dissemination of HBPM equipment among the hypertensive population is in the hands of public care centers, which should try to provide them to individual patients for the time interval recommended by guidelines (5-7 days before each visit). In conclusion, in low-income countries, the decision to use ABPM or HBPM would obviously be related to what is stated in the guidelines, but the availability, capacity and cultural level of patients must be taken into account in order to achieve an adequate blood pressure control. So, in developing countries the population would rather benefit from a simplification and reduction of the costs of the ABPM equipment to reach a wider and adequate control of hypertension.

**S14-Home Blood Pressure Monitoring in Clinical Research**

Average HBPM data, in particular, have been found to be more reproducible than OBP and even ABPM data in clinical trials, with a resulting reduction in the calculated sample size required for a comparative trial of the efficacy of antihypertensive drugs (16)

**General advantages of HBPM for clinical research** include the following: availability of multiple readings in the individual’s usual environment, obtained over several days, weeks, or even months. HBP values have a superior reproducibility and diagnostic accuracy compared to conventional OBP measurements, and show better correlation with early HMOD and cardiovascular events risk than OBP and similar to ABPM. HBPM is also more acceptable to patients than ABPM, and improves patients’ compliance with drug treatment. In patients on long-term dialysis, peridialysis BP measurements are unreliable but HBPM is an attractive alternative to ABPM (145), (146).

**HBPM may help improve study power and reduce sample size,** through a more accurate classification of the true baseline BP and drug treatment-induced BP changes, because of superior reproducibility of average BP values and absence of the placebo effect, of regression dilution bias (regression to the mean), and of observer bias (when electronic devices with automated memory are used), which typically affect clinical trials when OBP is employed (147).

**HBPM can be useful for chronotherapy studies** as it allows exploring the optimal time of drug administration in relation to BP levels, organ damage and adverse effects.

Also in a research setting the same methodological recommendations provided for clinical use of HBPM do apply, because optimal application of HBPM in clinical trials is of paramount importance for gathering reliable results. (16)

**Table S 1.** Prospective Studies of Home BP Monitoring and Cardiovascular Events. From Kario et al. (148) by permission


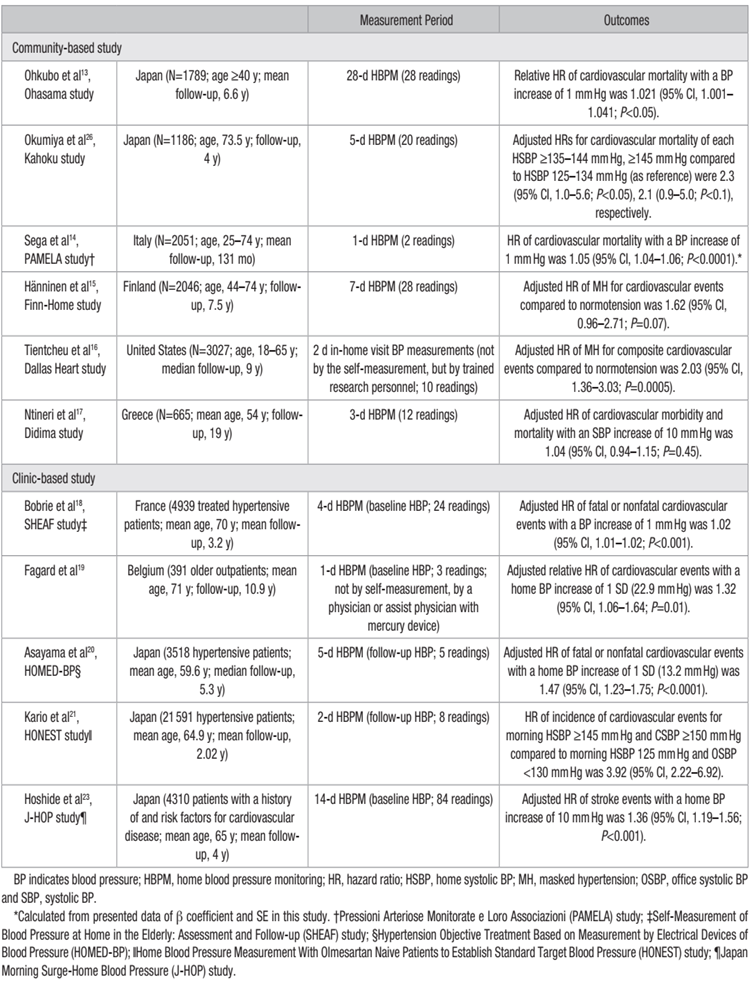


**References**

1. Whelton PK, Carey RM, Aronow WS, Casey DE, Jr., Collins KJ, Dennison Himmelfarb C, et al. 2017 ACC/AHA/AAPA/ABC/ACPM/AGS/APhA/ASH/ASPC/NMA/PCNA Guideline for the Prevention, Detection, Evaluation, and Management of High Blood Pressure in Adults: A Report of the American College of Cardiology/American Heart Association Task Force on Clinical Practice Guidelines. Hypertension. 2017.

2. Williams B, Mancia G, Spiering W, Agabiti Rosei E, Azizi M, Burnier M, et al. 2018 Practice Guidelines for the management of arterial hypertension of the European Society of Hypertension and the European Society of Cardiology: ESH/ESC Task Force for the Management of Arterial Hypertension. Journal of hypertension. 2018;36(12):2284-309.

3. Shimbo D, Artinian NT, Basile JN, Krakoff LR, Margolis KL, Rakotz MK, et al. Self-Measured Blood Pressure Monitoring at Home: A Joint Policy Statement From the American Heart Association and American Medical Association. Circulation. 2020;142(4):e42-e63.

4. Hypertension in adults: diagnosis and management. NICE guideline [NG136] [Available from: <https://www.nice.org.uk/guidance/ng136/chapter/recommendations>.

5. Lovibond K, Jowett S, Barton P, Caulfield M, Heneghan C, Hobbs FD, et al. Cost-effectiveness of options for the diagnosis of high blood pressure in primary care: a modelling study. Lancet. 2011;378(9798):1219-30.

6. Kario K, Imai Y, Kollias A, Niiranen TJ, Ohkubo T, McManus RJ, et al. Diagnostic value of home blood pressure: Springer 2019.

7. Sanchez RA, Boggia J, Penaherrera E, Barroso WS, Barbosa E, Villar R, et al. Ambulatory blood pressure monitoring over 24 h: A Latin American Society of Hypertension position paper-accessibility, clinical use and cost effectiveness of ABPM in Latin America in year 2020. J Clin Hypertens (Greenwich). 2020;22(4):527-43.

8. Villar R, Sanchez RA, Boggia J, Penaherrera E, Lopez J, Barroso WS, et al. Recommendations for home blood pressure monitoring in Latin American countries: A Latin American Society of Hypertension position paper. J Clin Hypertens (Greenwich). 2020;22(4):544-54.

9. Nerenberg KA, Zarnke KB, Leung AA, Dasgupta K, Butalia S, McBrien K, et al. Hypertension Canada's 2018 Guidelines for Diagnosis, Risk Assessment, Prevention, and Treatment of Hypertension in Adults and Children. Can J Cardiol. 2018;34(5):506-25.

10. Chatellier G, Day M, Bobrie G, Menard J. Feasibility study of N-of-1 trials with blood pressure self-monitoring in hypertension. Hypertension. 1995;25(2):294-301.

11. Chatellier G, Dutrey-Dupagne C, Vaur L, Zannad F, Genes N, Elkik F, et al. Home self blood pressure measurement in general practice. The SMART study. Self-measurement for the Assessment of the Response to Trandolapril. American journal of hypertension. 1996;9(7):644-52.

12. Sakuma M, Imai Y, Nagai K, Watanabe N, Sakuma H, Minami N, et al. Reproducibility of home blood pressure measurements over a 1-year period. American journal of hypertension. 1997;10(7 Pt 1):798-803.

13. Stergiou GS, Efstathiou SP, Argyraki CK, Gantzarou AP, Roussias LG, Mountokalakis TD. Clinic, home and ambulatory pulse pressure: comparison and reproducibility. Journal of hypertension. 2002;20(10):1987-93.

14. Kawabe H, Saito I. Which measurement of home blood pressure should be used for clinical evaluation when multiple measurements are made? Journal of hypertension. 2007;25(7):1369-74.

15. Guo QH, Cheng YB, Zhang DY, Wang Y, Huang QF, Sheng CS, et al. Comparison Between Home and Ambulatory Morning Blood Pressure and Morning Hypertension in Their Reproducibility and Associations With Vascular Injury. Hypertension. 2019;74(1):137-44.

16. Stergiou GS, Baibas NM, Gantzarou AP, Skeva, II, Kalkana CB, Roussias LG, et al. Reproducibility of home, ambulatory, and clinic blood pressure: implications for the design of trials for the assessment of antihypertensive drug efficacy. American journal of hypertension. 2002;15(2 Pt 1):101-4.

17. Uen S, Fimmers R, Brieger M, Nickenig G, Mengden T. Reproducibility of wrist home blood pressure measurement with position sensor and automatic data storage. BMC Cardiovasc Disord. 2009;9:20.

18. Imai Y, Otsuka K, Kawano Y, Shimada K, Hayashi H, Tochikubo O, et al. Japanese society of hypertension (JSH) guidelines for self-monitoring of blood pressure at home. Hypertens Res. 2003;26(10):771-82.

19. Imai Y, Kario K, Shimada K, Kawano Y, Hasebe N, Matsuura H, et al. The Japanese Society of Hypertension Guidelines for Self-monitoring of Blood Pressure at Home (Second Edition). Hypertens Res. 2012;35(8):777-95.

20. Park S, Buranakitjaroen P, Chen CH, Chia YC, Divinagracia R, Hoshide S, et al. Expert panel consensus recommendations for home blood pressure monitoring in Asia: the Hope Asia Network. Journal of human hypertension. 2018;32(4):249-58.

21. Viera AJ, Lin FC, Tuttle LA, Olsson E, Stankevitz K, Girdler SS, et al. Reproducibility of masked hypertension among adults 30 years or older. Blood pressure monitoring. 2014;19(4):208-15.

22. Verberk WJ, Kroon AA, Thien T, Lenders JW, van Montfrans GA, Smit AJ, et al. Prevalence of the white-coat effect at multiple visits before and during treatment. Journal of hypertension. 2006;24(12):2357-63.

23. Verberk WJ, Thien T, Kroon AA, Lenders JW, van Montfrans GA, Smit AJ, et al. Prevalence and persistence of masked hypertension in treated hypertensive patients. American journal of hypertension. 2007;20(12):1258-65.

24. Kawabe H, Saito I. Reproducibility of masked hypertension determined from morning and evening home blood pressure measurements over a 6-month period. Hypertens Res. 2007;30(9):845-51.

25. Shimamoto K, Ando K, Fujita T, Hasebe N, Higaki J, Horiuchi M, et al. The Japanese Society of Hypertension Guidelines for the Management of Hypertension (JSH 2014). Hypertens Res. 2014;37(4):253-390.

26. Umemura S, Arima H, Arima S, Asayama K, Dohi Y, Hirooka Y, et al. The Japanese Society of Hypertension Guidelines for the Management of Hypertension (JSH 2019). Hypertens Res. 2019;42(9):1235-481.

27. Stergiou GS, Bliziotis IA. Home blood pressure monitoring in the diagnosis and treatment of hypertension: a systematic review. American journal of hypertension. 2011;24(2):123-34.

28. Stergiou GS, Ntineri A. The optimal schedule for self-home blood pressure monitoring. Journal of hypertension. 2015;33(4):693-7.

29. Hodgkinson J, Mant J, Martin U, Guo B, Hobbs FD, Deeks JJ, et al. Relative effectiveness of clinic and home blood pressure monitoring compared with ambulatory blood pressure monitoring in diagnosis of hypertension: systematic review. BMJ. 2011;342:d3621.

30. Kang YY, Li Y, Huang QF, Song J, Shan XL, Dou Y, et al. Accuracy of home versus ambulatory blood pressure monitoring in the diagnosis of white-coat and masked hypertension. Journal of hypertension. 2015;33(8):1580-7.

31. Zhang L, Li Y, Wei FF, Thijs L, Kang YY, Wang S, et al. Strategies for classifying patients based on office, home, and ambulatory blood pressure measurement. Hypertension. 2015;65(6):1258-65.

32. Mancia G, Facchetti R, Bombelli M, Grassi G, Sega R. Long-term risk of mortality associated with selective and combined elevation in office, home, and ambulatory blood pressure. Hypertension. 2006;47(5):846-53.

33. Ntineri A, Niiranen TJ, McManus RJ, Lindroos A, Jula A, Schwartz C, et al. Ambulatory versus home blood pressure monitoring: frequency and determinants of blood pressure difference and diagnostic disagreement. Journal of hypertension. 2019;37(10):1974-81.

34. Stergiou GS, Kyriakoulis KG, McManus RJ, Andreadis EA, Jula A, Kollias A, et al. Phenotypes of masked hypertension: Isolated ambulatory, isolated home and dual masked hypertension. Journal of hypertension. 2020;38(2):218-23.

35. Anstey DE, Muntner P, Bello NA, Pugliese DN, Yano Y, Kronish IM, et al. Diagnosing Masked Hypertension Using Ambulatory Blood Pressure Monitoring, Home Blood Pressure Monitoring, or Both? Hypertension. 2018;72(5):1200-7.

36. Mancia G, Bombelli M, Brambilla G, Facchetti R, Sega R, Toso E, et al. Long-term prognostic value of white coat hypertension: an insight from diagnostic use of both ambulatory and home blood pressure measurements. Hypertension. 2013;62(1):168-74.

37. Satoh M, Asayama K, Kikuya M, Inoue R, Metoki H, Hosaka M, et al. Long-Term Stroke Risk Due to Partial White-Coat or Masked Hypertension Based on Home and Ambulatory Blood Pressure Measurements: The Ohasama Study. Hypertension. 2016;67(1):48-55.

38. Stergiou GS, Nasothimiou EG, Roussias LG. Morning hypertension assessed by home or ambulatory monitoring: different aspects of the same phenomenon? Journal of hypertension. 2010;28(9):1846-53.

39. Agarwal R, Pappas MK, Sinha AD. Masked Uncontrolled Hypertension in CKD. Journal of the American Society of Nephrology : JASN. 2016;27(3):924-32.

40. Kario K, Hasebe N, Okumura K, Yamashita T, Akao M, Atarashi H, et al. High prevalence of masked uncontrolled morning hypertension in elderly non-valvular atrial fibrillation patients: Home blood pressure substudy of the ANAFIE Registry. J Clin Hypertens (Greenwich). 2021;23(1):73-82.

41. Ishikawa J, Hoshide S, Eguchi K, Ishikawa S, Shimada K, Kario K, et al. Nighttime home blood pressure and the risk of hypertensive target organ damage. Hypertension. 2012;60(4):921-8.

42. Lin TT, Juang JJ, Lee JK, Tsai CT, Chen CH, Yu WC, et al. Comparison of Home and Ambulatory Blood Pressure Measurements in Association With Preclinical Hypertensive Cardiovascular Damage. J Cardiovasc Nurs. 2019;34(2):106-14.

43. Tsunoda S, Kawano Y, Horio T, Okuda N, Takishita S. Relationship between home blood pressure and longitudinal changes in target organ damage in treated hypertensive patients. Hypertens Res. 2002;25(2):167-73.

44. Niiranen TJ, Jula AM, Kantola IM, Karanko H, Reunanen A. Home-measured blood pressure is more strongly associated with electrocardiographic left ventricular hypertrophy than is clinic blood pressure: the Finn-HOME study. Journal of human hypertension. 2007;21(10):788-94.

45. Shibamiya T, Obara T, Ohkubo T, Shinki T, Ishikura K, Yoshida M, et al. Electrocardiographic abnormalities and home blood pressure in treated elderly hypertensive patients: Japan home versus office blood pressure measurement evaluation in the elderly (J-HOME-Elderly) study. Hypertens Res. 2010;33(7):670-7.

46. Hanninen MR, Niiranen TJ, Puukka PJ, Kesaniemi YA, Kahonen M, Jula AM. Target organ damage and masked hypertension in the general population: the Finn-Home study. Journal of hypertension. 2013;31(6):1136-43.

47. Kaihara T, Imaizumi Y, Eguchi K, Kario K. Maximum home blood pressure readings are associated with left atrial diameter in essential hypertensives. Journal of human hypertension. 2018;32(6):432-9.

48. Mancia G, Zanchetti A, Agabiti-Rosei E, Benemio G, De Cesaris R, Fogari R, et al. Ambulatory blood pressure is superior to clinic blood pressure in predicting treatment-induced regression of left ventricular hypertrophy. SAMPLE Study Group. Study on Ambulatory Monitoring of Blood Pressure and Lisinopril Evaluation. Circulation. 1997;95(6):1464-70.

49. Cuspidi C, Facchetti R, Quarti-Trevano F, Sala C, Tadic M, Grassi G, et al. Left ventricular mass and incident out-of-office hypertension in a general population. Journal of hypertension. 2020;38(4):633-40.

50. Siven SS, Niiranen TJ, Langen VL, Puukka PJ, Kantola IM, Jula AM. Home versus office blood pressure: longitudinal relations with left ventricular hypertrophy: the Finn-Home study. Journal of hypertension. 2017;35(2):266-71.

51. Matsui Y, Ishikawa J, Eguchi K, Shibasaki S, Shimada K, Kario K. Maximum value of home blood pressure: a novel indicator of target organ damage in hypertension. Hypertension. 2011;57(6):1087-93.

52. Fukuhara M, Arima H, Ninomiya T, Hata J, Hirakawa Y, Doi Y, et al. White-coat and masked hypertension are associated with carotid atherosclerosis in a general population: the Hisayama study. Stroke; a journal of cerebral circulation. 2013;44(6):1512-7.

53. Hara A, Ohkubo T, Kikuya M, Shintani Y, Obara T, Metoki H, et al. Detection of carotid atherosclerosis in individuals with masked hypertension and white-coat hypertension by self-measured blood pressure at home: the Ohasama study. Journal of hypertension. 2007;25(2):321-7.

54. Bliziotis IA, Destounis A, Stergiou GS. Home versus ambulatory and office blood pressure in predicting target organ damage in hypertension: a systematic review and meta-analysis. Journal of hypertension. 2012;30(7):1289-99.

55. Matsui Y, Eguchi K, Ishikawa J, Hoshide S, Shimada K, Kario K. Subclinical arterial damage in untreated masked hypertensive subjects detected by home blood pressure measurement. American journal of hypertension. 2007;20(4):385-91.

56. Agarwal R, Andersen MJ. Correlates of systolic hypertension in patients with chronic kidney disease. Hypertension. 2005;46(3):514-20.

57. Hata J, Fukuhara M, Sakata S, Arima H, Hirakawa Y, Yonemoto K, et al. White-coat and masked hypertension are associated with albuminuria in a general population: the Hisayama Study. Hypertens Res. 2017;40(11):937-43.

58. Agarwal R. Albuminuria and masked uncontrolled hypertension in chronic kidney disease. Nephrology, dialysis, transplantation : official publication of the European Dialysis and Transplant Association - European Renal Association. 2017;32(12):2058-65.

59. Kario K, Saito I, Kushiro T, Teramukai S, Ishikawa Y, Mori Y, et al. Home blood pressure and cardiovascular outcomes in patients during antihypertensive therapy: primary results of HONEST, a large-scale prospective, real-world observational study. Hypertension. 2014;64(5):989-96.

60. Hoshide S, Yano Y, Haimoto H, Yamagiwa K, Uchiba K, Nagasaka S, et al. Morning and Evening Home Blood Pressure and Risks of Incident Stroke and Coronary Artery Disease in the Japanese General Practice Population: The Japan Morning Surge-Home Blood Pressure Study. Hypertension. 2016;68(1):54-61.

61. Stergiou GS, Asayama K, Thijs L, Kollias A, Niiranen TJ, Hozawa A, et al. Prognosis of white-coat and masked hypertension: International Database of HOme blood pressure in relation to Cardiovascular Outcome. Hypertension. 2014;63(4):675-82.

62. Thakkar HV, Pope A, Anpalahan M. Masked Hypertension: A Systematic Review. Heart Lung Circ. 2020;29(1):102-11.

63. Cohen JB, Lotito MJ, Trivedi UK, Denker MG, Cohen DL, Townsend RR. Cardiovascular Events and Mortality in White Coat Hypertension: A Systematic Review and Meta-analysis. Annals of internal medicine. 2019;170(12):853-62.

64. Zhang DY, Guo QH, An DW, Li Y, Wang JG. A comparative meta-analysis of prospective observational studies on masked hypertension and masked uncontrolled hypertension defined by ambulatory and home blood pressure. Journal of hypertension. 2019;37(9):1775-85.

65. Fujiwara T, Yano Y, Hoshide S, Kanegae H, Kario K. Association of Cardiovascular Outcomes With Masked Hypertension Defined by Home Blood Pressure Monitoring in a Japanese General Practice Population. JAMA Cardiol. 2018;3(7):583-90.

66. Niiranen TJ, Asayama K, Thijs L, Johansson JK, Ohkubo T, Kikuya M, et al. Outcome-driven thresholds for home blood pressure measurement: international database of home blood pressure in relation to cardiovascular outcome. Hypertension. 2013;61(1):27-34.

67. Agarwal R, Andersen MJ. Prognostic importance of clinic and home blood pressure recordings in patients with chronic kidney disease. Kidney international. 2006;69(2):406-11.

68. Mokwatsi GG, Hoshide S, Kanegae H, Fujiwara T, Negishi K, Schutte AE, et al. Direct Comparison of Home Versus Ambulatory Defined Nocturnal Hypertension for Predicting Cardiovascular Events: The Japan Morning Surge-Home Blood Pressure (J-HOP) Study. Hypertension. 2020;76(2):554-61.

69. Tang O, Foti K, Miller ER, Appel LJ, Juraschek SP. Factors Associated With Physician Recommendation of Home Blood Pressure Monitoring and Blood Pressure in the US Population. American journal of hypertension. 2020;33(9):852-9.

70. Mengden T, Binswanger B, Spuhler T, Weisser B, Vetter W. The use of self-measured blood pressure determinations in assessing dynamics of drug compliance in a study with amlodipine once a day, morning versus evening. Journal of hypertension. 1993;11(12):1403-11.

71. Hodgkinson JA, Lee MM, Milner S, Bradburn P, Stevens R, Hobbs FR, et al. Accuracy of blood-pressure monitors owned by patients with hypertension (ACCU-RATE study): a cross-sectional, observational study in central England. Br J Gen Pract. 2020;70(697):e548-e54.

72. Kario K, Tomitani N, Iwashita C, Shiga T, Kanegae H. Simultaneous self-monitoring comparison of a supine algorithm-equipped wrist nocturnal home blood pressure monitoring device with an upper arm device. J Clin Hypertens (Greenwich). 2021.

73. Sharma M, Barbosa K, Ho V, Griggs D, Ghirmai T, Krishnan S, et al. Cuff-Less and Continuous Blood Pressure Monitoring: A Methodological Review. Technologies 2017;5(2):21.

74. Penaz J. Current photoelectric recording of blood flow through the

finger. . Cesk Fysiol 1975;24(4):349–52.

75. Imholz BP, Langewouters GJ, van Montfrans GA, Parati G, van Goudoever J, Wesseling KH, et al. Feasibility of ambulatory, continuous 24-hour finger arterial pressure recording. Hypertension. 1993;21(1):65-73.

76. Thomas SS, Nathan V, Chengzhi Z, Akinbola E, Aroul AL, Philipose L, et al. BioWatch - a wrist watch based signal acquisition system for physiological signals including blood pressure. Conf Proc IEEE Eng Med Biol Soc. 2014;2014:2286-9.

77. Zhang Q, Shi Y, Teng D, Dinh A, Ko SB, Chen L, et al. Pulse transit time-based blood pressure estimation using hilbert-huang transform. Conf Proc IEEE Eng Med Biol Soc. 2009;2009:1785-8.

78. Mukkamala R, Hahn JO, Inan OT, Mestha LK, Kim CS, Toreyin H, et al. Toward Ubiquitous Blood Pressure Monitoring via Pulse Transit Time: Theory and Practice. IEEE Trans Biomed Eng. 2015;62(8):1879-901.

79. Jeong I, Wood J, Finkelstein J. Using individualized pulse transit time calibration to monitor blood pressure during exercise. Stud Health Technol Inform. 2013;190:39-41.

80. Yoon Y, Cho JH, Yoon G. Non-constrained blood pressure monitoring using ECG and PPG for personal healthcare. J Med Syst. 2009;33(4):261-6.

81. Feng J, Huang Z, Zhou C, Ye X. Study of continuous blood pressure estimation based on pulse transit time, heart rate and photoplethysmography-derived hemodynamic covariates. Australas Phys Eng Sci Med. 2018;41(2):403-13.

82. Lass J, Meigas K, Karai D, Kattai R, Kaik J, Rossmann M. Continuous blood pressure monitoring during exercise using pulse wave transit time measurement. Conf Proc IEEE Eng Med Biol Soc. 2004;2004:2239-42.

83. Lazazzera R, Belhaj Y, Carrault G. A New Wearable Device for Blood Pressure Estimation Using Photoplethysmogram. Sensors (Basel). 2019;19(11).

84. Sola J, Proenca M, Ferrario D, Porchet JA, Falhi A, Grossenbacher O, et al. Noninvasive and Nonocclusive Blood Pressure Estimation Via a Chest Sensor. IEEE Trans Biomed Eng. 2013;60(12):3505-13.

85. Matsumura K, Rolfe P, Toda S, Yamakoshi T. Cuffless blood pressure estimation using only a smartphone. Sci Rep. 2018;8(1):7298.

86. Salvi P, Grillo A, Parati G. Noninvasive estimation of central blood pressure and analysis of pulse waves by applanation tonometry. Hypertens Res. 2015;38(10):646-8.

87. Salvi P, Safar ME, Parati G. Arterial applanation tonometry: technical aspects relevant for its daily clinical use. Journal of hypertension. 2013;31(3):469-71.

88. Salvi P, Parati G. Methodological aspects in the measurement of pulse wave velocity by means of applanation tonometry. Journal of hypertension. 2013;31(1):35-8.

89. Agnoletti D, Zhang Y, Salvi P, Borghi C, Topouchian J, Safar ME, et al. Pulse pressure amplification, pressure waveform calibration and clinical applications. Atherosclerosis. 2012;224(1):108-12.

90. Bilo G, Zorzi C, Ochoa Munera JE, Torlasco C, Giuli V, Parati G. Validation of the Somnotouch-NIBP noninvasive continuous blood pressure monitor according to the European Society of Hypertension International Protocol revision 2010. Blood pressure monitoring. 2015;20(5):291-4.

91. Persell SD, Peprah YA, Lipiszko D, Lee JY, Li JJ, Ciolino JD, et al. Effect of Home Blood Pressure Monitoring via a Smartphone Hypertension Coaching Application or Tracking Application on Adults With Uncontrolled Hypertension: A Randomized Clinical Trial. JAMA Netw Open. 2020;3(3):e200255.

92. Niiranen TJ, McManus RJ, Ohkubo T, Stergiou GS. Home blood pressure monitoring schedule. Updates in Hypertension and Cardiovascular Protection; Home Blood Pressure Monitoring: Springer 2019. p. 55-62.

93. Stergiou GS, Skeva, II, Zourbaki AS, Mountokalakis TD. Self-monitoring of blood pressure at home: how many measurements are needed? Journal of hypertension. 1998;16(6):725-31.

94. Niiranen TJ, Asayama K, Thijs L, Johansson JK, Hara A, Hozawa A, et al. Optimal number of days for home blood pressure measurement. American journal of hypertension. 2015;28(5):595-603.

95. Johansson JK, Niiranen TJ, Puukka PJ, Jula AM. Optimal schedule for home blood pressure monitoring based on a clinical approach. Journal of hypertension. 2010;28(2):259-64.

96. Bello NA, Schwartz JE, Kronish IM, Oparil S, Anstey DE, Wei Y, et al. Number of Measurements Needed to Obtain a Reliable Estimate of Home Blood Pressure: Results From the Improving the Detection of Hypertension Study. J Am Heart Assoc. 2018;7(20):e008658.

97. Niiranen TJ, Johansson JK, Reunanen A, Jula AM. Optimal schedule for home blood pressure measurement based on prognostic data: the Finn-Home Study. Hypertension. 2011;57(6):1081-6.

98. Hodgkinson JA, Stevens R, Grant S, Mant J, Bray EP, Hobbs FDR, et al. Schedules for Self-monitoring Blood Pressure: A Systematic Review. American journal of hypertension. 2019;32(4):350-64.

99. Eguchi K, Kuruvilla S, Ogedegbe G, Gerin W, Schwartz JE, Pickering TG. What is the optimal interval between successive home blood pressure readings using an automated oscillometric device? Journal of hypertension. 2009;27(6):1172-7.

100. Staessen JA, Den Hond E, Celis H, Fagard R, Keary L, Vandenhoven G, et al. Antihypertensive treatment based on blood pressure measurement at home or in the physician's office: a randomized controlled trial. JAMA. 2004;291(8):955-64.

101. Verberk WJ, Kroon AA, Kessels AG, Dirksen C, Nelemans PJ, Lenders JW, et al. Home versus Office blood pressure MEasurements: Reduction of Unnecessary treatment Study: rationale and study design of the HOMERUS trial. Blood Press. 2003;12(5-6):326-33.

102. Verberk WJ, Kroon AA, Lenders JW, Kessels AG, van Montfrans GA, Smit AJ, et al. Self-measurement of blood pressure at home reduces the need for antihypertensive drugs: a randomized, controlled trial. Hypertension. 2007;50(6):1019-25.

103. Stergiou GS, Karpettas N, Destounis A, Tzamouranis D, Nasothimiou E, Kollias A, et al. Home blood pressure monitoring alone vs. combined clinic and ambulatory measurements in following treatment-induced changes in blood pressure and organ damage. American journal of hypertension. 2014;27(2):184-92.

104. Aparicio LS, Thijs L, Boggia J, Jacobs L, Barochiner J, Odili AN, et al. Defining thresholds for home blood pressure monitoring in octogenarians. Hypertension. 2015;66(4):865-73.

105. McManus RJ, Mant J, Franssen M, Nickless A, Schwartz C, Hodgkinson J, et al. Efficacy of self-monitored blood pressure, with or without telemonitoring, for titration of antihypertensive medication (TASMINH4): an unmasked randomised controlled trial. Lancet. 2018;391(10124):949-59.

106. Monahan M, Jowett S, Nickless A, Franssen M, Grant S, Greenfield S, et al. Cost-Effectiveness of Telemonitoring and Self-Monitoring of Blood Pressure for Antihypertensive Titration in Primary Care (TASMINH4). Hypertension. 2019;73(6):1231-9.

107. Zarnke KB, Feagan BG, Mahon JL, Feldman RD. A randomized study comparing a patient-directed hypertension management strategy with usual office-based care. American journal of hypertension. 1997;10(1):58-67.

108. McManus RJ, Mant J, Bray EP, Holder R, Jones MI, Greenfield S, et al. Telemonitoring and self-management in the control of hypertension (TASMINH2): a randomised controlled trial. Lancet. 2010;376(9736):163-72.

109. O'Brien E, Dolan E, Stergiou GS. Achieving reliable blood pressure measurements in clinical practice: It's time to meet the challenge. J Clin Hypertens (Greenwich). 2018;20(7):1084-8.

110. Myers MG, Asmar R, Staessen JA. Office blood pressure measurement in the 21st century. J Clin Hypertens (Greenwich). 2018;20(7):1104-7.

111. O'Brien E, White WB, Parati G, Dolan E. Ambulatory blood pressure monitoring in the 21st century. J Clin Hypertens (Greenwich). 2018;20(7):1108-11.

112. Stergiou GS, Kario K, Kollias A, McManus RJ, Ohkubo T, Parati G, et al. Home blood pressure monitoring in the 21st century. J Clin Hypertens (Greenwich). 2018;20(7):1116-21.

113. Stergiou G, Kollias A, Parati G, O'Brien E. Office Blood Pressure Measurement: The Weak Cornerstone of Hypertension Diagnosis. Hypertension. 2018;71(5):813-5.

114. O'Brien E, Kario K, Staessen JA, de la Sierra A, Ohkubo T. Patterns of ambulatory blood pressure: clinical relevance and application. J Clin Hypertens (Greenwich). 2018;20(7):1112-5.

115. O'Brien E, Stergiou GS, Turner MJ. The quest for accuracy of blood pressure measuring devices. J Clin Hypertens (Greenwich). 2018;20(7):1092-5.

116. Parati G, Stergiou GS, Asmar R, Bilo G, de Leeuw P, Imai Y, et al. European Society of Hypertension guidelines for blood pressure monitoring at home: a summary report of the Second International Consensus Conference on Home Blood Pressure Monitoring. Journal of hypertension. 2008;26(8):1505-26.

117. Parati G, Stergiou G, O'Brien E, Asmar R, Beilin L, Bilo G, et al. European Society of Hypertension practice guidelines for ambulatory blood pressure monitoring. Journal of hypertension. 2014;32(7):1359-66.

118. O’Brien E, de la Sierra A, McManus RJ, Mihailidou A, Muntner P, Myers MG, et al. Home Versus Ambulatory Blood Pressure Monitoring. In: al. Se, editor. Updates in Hypertension and Cardiovascular Protection; Home Blood Pressure Monitoring: Springer 2019.

119. Stergiou GS, Salgami EV, Tzamouranis DG, Roussias LG. Masked hypertension assessed by ambulatory blood pressure versus home blood pressure monitoring: is it the same phenomenon? American journal of hypertension. 2005;18(6):772-8.

120. Sega R, Trocino G, Lanzarotti A, Carugo S, Cesana G, Schiavina R, et al. Alterations of cardiac structure in patients with isolated office, ambulatory, or home hypertension: Data from the general population (Pressione Arteriose Monitorate E Loro Associazioni [PAMELA] Study). Circulation. 2001;104(12):1385-92.

121. Martinez MA, Sancho T, Garcia P, Moreno P, Rubio JM, Palau FJ, et al. Home blood pressure in poorly controlled hypertension: relationship with ambulatory blood pressure and organ damage. Blood pressure monitoring. 2006;11(4):207-13.

122. Fagard RH, Van Den Broeke C, De Cort P. Prognostic significance of blood pressure measured in the office, at home and during ambulatory monitoring in older patients in general practice. Journal of human hypertension. 2005;19(10):801-7.

123. Mule G, Caimi G, Cottone S, Nardi E, Andronico G, Piazza G, et al. Value of home blood pressures as predictor of target organ damage in mild arterial hypertension. J Cardiovasc Risk. 2002;9(2):123-9.

124. Verdecchia P, Clement D, Fagard R, Palatini P, Parati G. Blood Pressure Monitoring. Task force III: Target-organ damage, morbidity and mortality. Blood pressure monitoring. 1999;4(6):303-17.

125. Sega R, Facchetti R, Bombelli M, Cesana G, Corrao G, Grassi G, et al. Prognostic value of ambulatory and home blood pressures compared with office blood pressure in the general population: follow-up results from the Pressioni Arteriose Monitorate e Loro Associazioni (PAMELA) study. Circulation. 2005;111(14):1777-83.

126. Stergiou GS, Siontis KC, Ioannidis JP. Home blood pressure as a cardiovascular outcome predictor: it's time to take this method seriously. Hypertension. 2010;55(6):1301-3.

127. Ntineri A, Kalogeropoulos PG, Kyriakoulis KG, Aissopou EK, Thomopoulou G, Kollias A, et al. Prognostic value of average home blood pressure and variability: 19-year follow-up of the Didima study. Journal of hypertension. 2018;36(1):69-76.

128. Agarwal R, Satyan S, Alborzi P, Light RP, Tegegne GG, Mazengia HS, et al. Home blood pressure measurements for managing hypertension in hemodialysis patients. American journal of nephrology. 2009;30(2):126-34.

129. Agarwal R. Managing hypertension using home blood pressure monitoring among haemodialysis patients--a call to action. Nephrology, dialysis, transplantation : official publication of the European Dialysis and Transplant Association - European Renal Association. 2010;25(6):1766-71.

130. Lins RL, Elseviers M, Rogiers P, Van Hoeyweghen RJ, De Raedt H, Zachee P, et al. Importance of volume factors in dialysis related hypertension. Clinical nephrology. 1997;48(1):29-33.

131. Agarwal R, Brim NJ, Mahenthiran J, Andersen MJ, Saha C. Out-of-hemodialysis-unit blood pressure is a superior determinant of left ventricular hypertrophy. Hypertension. 2006;47(1):62-8.

132. Agarwal R. Blood pressure and mortality among hemodialysis patients. Hypertension. 2010;55(3):762-8.

133. da Silva GV, de Barros S, Abensur H, Ortega KC, Mion D, Jr., Cochrane Renal Group Prospective Trial Register CRG. Home blood pressure monitoring in blood pressure control among haemodialysis patients: an open randomized clinical trial. Nephrology, dialysis, transplantation : official publication of the European Dialysis and Transplant Association - European Renal Association. 2009;24(12):3805-11.

134. Agarwal R, Andersen MJ, Light RP. Location not quantity of blood pressure measurements predicts mortality in hemodialysis patients. American journal of nephrology. 2008;28(2):210-7.

135. Alborzi P, Patel N, Agarwal R. Home blood pressures are of greater prognostic value than hemodialysis unit recordings. Clinical journal of the American Society of Nephrology : CJASN. 2007;2(6):1228-34.

136. Liu W, Ye H, Tang B, Sun Z, Wen P, Wu W, et al. Comparison of 44-hour and fixed 24-hour ambulatory blood pressure monitoring in dialysis patients. J Clin Hypertens (Greenwich). 2014;16(1):63-9.

137. Agarwal R, Light RP. Chronobiology of arterial hypertension in hemodialysis patients: implications for home blood pressure monitoring. American journal of kidney diseases : the official journal of the National Kidney Foundation. 2009;54(4):693-701.

138. Agarwal R, Peixoto AJ, Santos SF, Zoccali C. Pre- and postdialysis blood pressures are imprecise estimates of interdialytic ambulatory blood pressure. Clinical journal of the American Society of Nephrology : CJASN. 2006;1(3):389-98.

139. Fujiwara T, Hoshide S, Kanegae H, Kario K. Cardiovascular Event Risks Associated With Masked Nocturnal Hypertension Defined by Home Blood Pressure Monitoring in the J-HOP Nocturnal Blood Pressure Study. Hypertension. 2020;76(1):259-66.

140. Juhanoja EP, Johansson JK, Puukka PJ, Jula AM, Niiranen TJ. Optimal Schedule for Assessing Home BP Variability: The Finn-Home Study. American journal of hypertension. 2018;31(6):715-25.

141. Matsui Y, O'Rourke MF, Hoshide S, Ishikawa J, Shimada K, Kario K. Combined effect of angiotensin II receptor blocker and either a calcium channel blocker or diuretic on day-by-day variability of home blood pressure: the Japan Combined Treatment With Olmesartan and a Calcium-Channel Blocker Versus Olmesartan and Diuretics Randomized Efficacy Study. Hypertension. 2012;59(6):1132-8.

142. Ushigome E, Matsumoto S, Oyabu C, Ushigome H, Yokota I, Hasegawa G, et al. Olmesartan with azelnidipine versus with trichlormethiazide on home blood pressure variability in patients with type II diabetes mellitus. Journal of the American Society of Hypertension : JASH. 2017;11(3):140-7.

143. Miyoshi T, Suetsuna R, Tokunaga N, Kusaka M, Tsuzaki R, Koten K, et al. Effect of Azilsartan on Day-to-Day Variability in Home Blood Pressure: A Prospective Multicenter Clinical Trial. J Clin Med Res. 2017;9(7):618-23.

144. Asayama K, Ohkubo T, Hanazawa T, Watabe D, Hosaka M, Satoh M, et al. Does Antihypertensive Drug Class Affect Day-to-Day Variability of Self-Measured Home Blood Pressure? The HOMED-BP Study. J Am Heart Assoc. 2016;5(3):e002995.

145. Agarwal R, Sinha AD, Pappas MK, Abraham TN, Tegegne GG. Hypertension in hemodialysis patients treated with atenolol or lisinopril: a randomized controlled trial. Nephrology, dialysis, transplantation : official publication of the European Dialysis and Transplant Association - European Renal Association. 2014;29(3):672-81.

146. Agarwal R, Alborzi P, Satyan S, Light RP. Dry-weight reduction in hypertensive hemodialysis patients (DRIP): a randomized, controlled trial. Hypertension. 2009;53(3):500-7.

147. Mengden T, Binswanger B, Weisser B, Vetter W. An evaluation of self-measured blood pressure in a study with a calcium-channel antagonist versus a beta-blocker. American journal of hypertension. 1992;5(3):154-60.

148. Kario K, Shimbo D, Hoshide S, Wang JG, Asayama K, Ohkubo T, et al. Emergence of Home Blood Pressure-Guided Management of Hypertension Based on Global Evidence. Hypertension. 2019:HYPERTENSIONAHA11912630.
